# Comparative Proteomic Study of the Antiproliferative Activity of Frog Host-Defence Peptide Caerin 1.9 and Its Additive Effect with Caerin 1.1 on TC-1 Cells Transformed with HPV16 E6 and E7

**DOI:** 10.1155/2018/7382351

**Published:** 2018-05-13

**Authors:** Guoying Ni, Di Liang, Scott F. Cummins, Shelley F. Walton, Shu Chen, Yuejian Wang, Kate Mounsey, Ming Q. Wei, Jianwei Yuan, Xuan Pan, Xiaosong Liu, Tianfang Wang

**Affiliations:** ^1^The First Affiliated Hospital, School of Clinical Medicine, Guangdong Pharmaceutical University, Guangzhou 510080, China; ^2^Genecology Research Centre, University of the Sunshine Coast, Maroochydore, QLD 4558, Australia; ^3^Inflammation and Healing Research Cluster, School of Health and Sport Sciences, University of Sunshine Coast, Maroochydore, QLD 4558, Australia; ^4^Cancer Research Institute, First People's Hospital of Foshan, Foshan, Guangdong 528000, China; ^5^School of Medical Science, Griffith Health Institute, Griffith University, Gold Coast, QLD 4222, Australia

## Abstract

Caerin is a family of peptides isolated from the glandular secretion of Australian tree frogs, the genus* Litoria*, and has been previously shown to have anticancer activity against several cancer cells. In this work, we used two host-defence peptides, caerin 1.1 and caerin 1.9, to investigate their ability to inhibit a murine derived TC-1 cell transformed with human papillomavirus 16 E6 and E7 growth* in vitro*. Caerin 1.9 inhibits TC-1 cell proliferation, although inhibition is more pronounced when applied in conjunction with caerin 1.1. To gain further insights into the antiproliferative mechanisms of caerin 1.9 and its additive effect with caerin 1.1, we used a proteomics strategy to quantitatively examine (i) the changes in the protein profiles of TC-1 cells and (ii) the excretory-secretory products of TC-1 cells following caerin peptides treatment. Caerin 1.9 treatment significantly altered the abundance of several immune-related proteins and related pathways, such as the Tec kinase and ILK signalling pathways, as well as the levels of proinflammatory cytokines and chemokines. In conclusion, caerin peptides inhibit TC-1 cell proliferation, associated with modification in signalling pathways that would change the tumour microenvironment which is normally immune suppressive.

## 1. Introduction

Cervical cancer is the third most diagnosed cancer in woman and is associated with high risk human papillomavirus (HPV) infection, especially HPV subtypes 16 and 18 [1]. Targeted therapies are currently under intensive investigation. These therapies target tumour cells either directly or via interfering with the tumour microenvironment (TME) [2].

Therapeutic vaccines target tumour cells without harming normal tissues or organs. However, to date therapeutic vaccines have only shown efficacies in some but not all patients [3]. One of the key problems that limit the effectiveness of therapeutic vaccines is the immune suppressive TME, which may prevent the migration of tumour killing effector cells to the tumour sites or the killing of the tumour cells [3, 4]. Targeting the TME coupled with therapeutic vaccines may provide an effective way for better treatment of cancers.

Innate immune polypeptides have been shown to overcome the immune suppressive TME via a unique cancer cell killing mechanism possibly involving cell membrane lysis [5–9]. These peptides were initially discovered due to their function in clearing bacteria, while some were also highly active against cancer cells but not normal mammalian cells [10–13]. It is not known and worthy of investigation to determine if these peptides are able to directly kill tumour cells and at the same time improve the immune suppressive TME. If confirmed, the peptides are potential drug candidates given that peptides are usually easy to synthesise at a relatively large scale and may have minimal side effects [14–16].

During the last three decades, more than 200 host-defence peptides have been isolated and identified from skin secretions of Australian frogs and toads. Many of these peptides show antimicrobial and/or neuropeptide-type activities [17–19] and inhibit the aggregation of Amyloid beta 42, which is the major precursor of the extracellular fibrillar deposits of Alzheimer's disease [20]. The caerin 1 peptides have previously been shown to be potent membrane-active peptides and to stop the formation of nitric oxide by neuronal nitric oxide synthase [21, 22]. It has been previously reported that caerin 1.1 (^1^GLLSVLGSV^10^AKHVLPHVLP^20^HVVPVIAEHL-NH_2_) has an anticancer effect against a number of human cancer cell lines (including leukaemia, lung, colon, CNS, melanoma, ovarian, renal, prostate, and breast cancers) [21, 22]. The caerin 1.9 peptide (^1^GLFGVLGSI^10^AKHVLPHVVP^20^VIAEKL-NH_2_) has reported antimicrobial activity against a wide spectrum of Gram-positive and Gram-negative microbial strains [23]. It has also been found that caerin 1.1 and caerin 1.9 inhibit HIV-infected T cells within minutes after exposure at concentrations nontoxic to target cells and inhibit the transfer of HIV from dendritic cells (DCs) to T cells [24].

The TC-1 cell is a lung cancer cell line transformed with human papilloma virus early genes E6/E7, which are oncoproteins expressed in cervical cancer cells and sufficient to maintain the malignant status. The TC-1 cell line is used as surrogate for cervical cancer study in mice and has been widely used as a tumour model. The effect of caerin peptides on TC-1 cells and the molecular mechanism underlying the potential interaction have not been reported. This work describes the use of* in vitro* assays and quantitative proteomic methods to study the effect upon the proliferation of the cervical cancer cell TC-1 by caerin 1.9 and the potential additive effect when caerin 1.9 is applied in conjunction with caerin 1.1. The objectives of the study were to identify (i) changes in the profiles of proteins in TC-1 cells and excretory-secretory proteins (ESPs), following treatments of caerin 1.9 and the caerin 1.1/1.9 mixture, and (ii) quantitative proteomic differences between untreated and treated conditions to gain insights into the antiproliferative mechanisms induced by caerin 1.9. To our knowledge, this is the first proteomic study on the bioactivity of caerin peptides on cervical cancer using high-resolution mass spectrometry profiling, iTRAQ labelling, and label-free quantitation.

## 2. Materials and Methods

### 2.1. Chemicals

Trifluoroacetic acid (TFA), methanol, acetonitrile (ACN), formic acid, NH_4_HCO_3_, urea, dithiothreitol (DTT), iodoacetamide (IAA), sodium pyruvate, L-glutamine, G418, and nonessential amino acid solution were obtained from Sigma-Aldrich (St. Louis, MO). Trypsin (Mass Spec grade V5280) was purchased from Promega (Madison, WI). Ultrapure water was prepared by MilliQ water purification system (Millipore, Bedford, MA). Isobaric tag for relative and absolute quantitation (iTRAQ) 4-plex kit was purchased from AB SCIEX (Concord, Canada).

### 2.2. Cell Line, Cell Culture, and Peptide Synthesis

A murine TC-1 cell line was purchased from Shanghai Institutes for Cell Resource Centre, Chinese Academy of Sciences, and cultured following the protocols in the product sheets. Briefly, TC-1 cells were cultured in complete RPMI 1640 media (GIBCO) supplemented with 10% heat inactivated fetal calf serum (FCS, GIBCO), 100 U of penicillin/mL and 100 *μ*g of streptomycin/mL (GIBCO), 0.2 mM nonessential amino acid solution, 1.0 mM sodium pyruvate, 2 mM L-glutamine, and 0.4 mg/mL G418 and were cultured at 37°C with 5% CO_2_.

Human HMC, HeLa cell lines were purchased from the Shanghai Institutes for Biological Sciences, Chinese Academy of Sciences. The cell lines were cultured in complete RPMI 1640 media (GIBCO) supplemented with 10% heat inactivated fetal calf serum (FCS, GIBCO), 100 U of penicillin/mL and 100 *μ*g of streptomycin/mL (GIBCO), 0.2 mM nonessential amino acid solution, 1.0 mM sodium pyruvate, and 2 mM L-glutamine, 0.4 mg/mL G418 and were cultured at 37°C with 5% CO_2_.

Human NP69 cell line was purchased from the Shanghai Institutes for Biological Sciences, Chinese Academy of Sciences. The cell line NP69 was grown in Keratinocyte-SFM (GIBCO) supplemented with 10% heat inactivated fetal calf serum (FCS, GIBCO), 100 U of penicillin/mL and 100 *μ*g of streptomycin/mL (GIBCO), 0.2 ng/mL rEGF (GIBCO), and 30 *μ*g/m1 BPE (GIBCO) in humidified atmosphere of 5% CO_2_ at 37°C.

Caerin 1.1 (GLLSVLGSVAKHVLPHVLPHVVPVIAEHL-NH_2_), caerin 1.9 (GLFGVLGSIAKHVLPHVVPVIAEKL-NH_2_), and P3 (GTELPSPPSVWFEAEFK-OH) were synthesised by Mimotopes (Melbourne, Australia). The purity of the peptides was >95% as determined by reverse-phase HPLC, done at Mimotopes.

### 2.3. MTT Assay

Cell proliferation was determined by an MTT assay (ATCC, USA) following manufacturer instructions. Briefly, TC-1 and NP69 cells were cultured separately in flat bottomed 96 well plates. Approximately 0–15 *μ*g of peptides were added to 5 × 10^3^ of TC-1 cells or NP69 cells and cultured overnight at 37°C with 5% CO_2_. Each treatment was performed in triplicate. Ten microliters of MTT stock solution were added and cultured for another 4 h, before 100 *μ*l of DMSO was added to stop the experiment. Results were analysed with an ELISA plate reader (BioTek, USA) at 450 nm according to the manufacturer's protocol.

### 2.4. Flow Cytometric Analysis

FITC-labelled caerin 1.9 and P3 were added to 5 × 10^5^ TC-1 cells at different concentrations (10, 5, and 1 *μ*g/mL), 24 hr. After extensive washes with PBS (phosphate-buffered saline) containing 2% FCS, the cells were acquired on BD FACSCalibur (BD, USA). Flow cytometry data was analysed by FlowJo (Tristar, USA).

### 2.5. Cell Lysis and Sample Preparation for LC-MS/MS

TC-1 cells treated with caerin 1.9 or/and caerin 1.1 were collected. The concentration of the peptides is 5 *μ*g/ml. Cell pellets were washed with 1 mL of cold PBS and counted. After being counted, 1 × 10^6^ cells were lysed with 300 *μ*l of lysis buffer (8 M urea, 0.8 M NH_4_HCO_3_, and pH 8.0) supplemented with 10 *μ*l of protease inhibitor cocktail (80-6501-23, GE Healthcare, Little Chalfont, UK) to prevent protein degradation. The samples were then sonicated for 30 min on ice and then centrifuged at 12,000 ×g at 4°C for 15 min. The supernatants were collected, and protein concentration in the cell lysates was measured using a NanoDrop 2000 (Thermo Fisher Scientific, Bremen, Germany). Then, 500 *μ*g of proteins in 100 *μ*l lysis buffer was reduced with 5 *μ*l of 100 mM DTT for 1 h at 37°C and subsequently alkylated with 20 *μ*l of 100 mM IAA for 1 h at room temperature (RT) in the dark, followed by the incubation with the addition of 20 *μ*L of 100 mM DTT at RT for 45 min. The urea concentration was reduced by diluting the reaction mixture with 775 *μ*l MilliQ water, then the proteins digested with sequencing grade modified trypsin at 1 : 50 enzyme‐to‐substrate ratio. After 4 h of digestion at 37°C, samples were diluted 1 : 4 with 50 mM NH_4_HCO_3_ and 1 mM CaCl_2_ and another aliquot of the same amount of trypsin was added to the samples and further incubated at room temperature overnight (~16 h).

The digested samples were then acidified with 10% FA to pH < 3. Tryptic peptides were desalted on Sep-Pak C18 columns (Waters, Milford, MA) and dried using Speed-Vac. Peptides (100 *μ*g) from three samples were dissolved in 30 *μ*L of 0.5 M triethylammonium bicarbonate, pH 8.5 solution, and mixed with 1 units of iTRAQ reagent that was dissolved freshly in 70 *μ*L of ethanol. Channel 114 was used for labelling the reference (untreated), 116 for labelling caerin 1.9, and 117 l for labelling 1.1 plus 1.9 treated cell samples. After 1 h incubation at RT, 300 *μ*L of water was added and incubated for 30 min at RT to stop the reaction and hydrolyze the unreacted iTRAQ reagents. Peptides labelled by different iTRAQ reagents were then mixed and concentrated to ~200 *μ*L, were desalted on C18 SPE columns, and dried and stored at −20°C.

### 2.6. Cell Culture Supernatant Collection and Preparation for LC-MS/MS

To study change in cellular communication in response to peptide treatment, excretory/secretory proteins (ESPs) were analysed. Approximately 1 mL cell culture supernatant was collected and centrifuged at 2,000 ×g, 4°C for 20 min. The supernatant was transferred (0.5 cm of liquid above the pellet was left to ensure no contamination) into Amicon Ultra-2 mL 30K MWCO centrifugal filter (Millipore, Billerica, MA) using a pipette and centrifuged for 30 min at 7,500 ×g, 4°C. The filtrate was collected and lyophilised and subjected to in-solution digestion as described above. Tryptic peptides were desalted on Sep-Pak C18 columns, dried, and resuspended in 0.5% FA for LC-MS/MS.

### 2.7. Nano-LC Tandem Triple TOF-MS/MS Analyses

The iTRAQ labelled peptides were resuspended in 25 *μ*L 0.5% FA in MilliQ water and analysed by LC-MS/MS on a Shimadzu Prominance Nano HPLC (Kyoto, Japan) coupled to a Triple TOF 5600 mass spectrometer (AB SCIEX, Concord, Canada) equipped with a nanoelectrospray ion source. Ten microliters of each extract was injected onto a 50 mm × 300 *μ*m C18 trap column (Agilent Technologies, Sydney, Australia) at 30 *μ*L/min. The samples were desalted on the trap column for 5 minutes using 0.1% formic acid (aq) at 30 *μ*L/min. The trap column was then placed in-line with the analytical nano-HPLC column, a 150 mm × 75 *μ*m 300SBC18, 3.5 um (Agilent Technologies, Australia) for mass spectrometry analysis. Linear gradients of 1–60% solvent B over 170 min (60 min was used for the separation of peptides derived from cell supernatant) at 300 nL/min flow rate, followed by a steeper gradient from 60% to 80% solvent B in 5 min, were used for peptide elution. Solvent B was held at 80% for 5 min for washing the column and returned to 1% solvent B for equilibration prior to the next sample injection. Solvent A consisted of 0.1% formic acid (aq) and solvent B contained 90/10 acetonitrile/0.1% formic acid (aq). The ionspray voltage was set to 2400 V, declustering potential (DP) 100 V, curtain gas flow 25, nebuliser gas 1 (GS1) 12, and interface heater at 150°C. The mass spectrometer acquired 500 ms full scan TOF-MS data followed by 20 by 50 ms full scan product ion data in an Information Dependant Acquisition, IDA mode. Full scan TOF-MS data was acquired over the mass range 350–1800 and for product ion ms/ms 100–1800. Ions observed in the TOF-MS scan exceeding a threshold of 100 counts and a charge state of +2 to +5 were set to trigger the acquisition of product ion, ms/ms spectra of the resultant 20 most intense ions. The data was acquired and processed using Analyst TF 1.5.1 software (AB SCIEX, Concord, Canada). Biological triplicates were used for both cell and supernatant analysis.

### 2.8. Cell and Supernatant Protein Identification and Quantification

The LC-MS/MS data were imported to the PEAKS studio (Bioinformatics Solutions Inc., Waterloo, ON, Canada, version 7.0) with the assistance of MS Data Converter (Beta 1.3, https://sciex.com/software-downloads-x2110). The database of human proteome used for the analysis in this study was downloaded from Uniprot (http://www.uniprot.org/proteomes/UP000005640) in May 2016, which contains 70,613 entries. De novo sequencing of peptides, database search, and characterising specific PTMs were used to analyse the raw data; false discovery rate (FDR) was set to ≤1%, and [−10*∗*log⁡(*P*)] was calculated accordingly where *P* is the probability that an observed match is a random event. The PEAKS used the following parameters: (i) precursor ion mass tolerance, 0.1 Da; (ii) fragment ion mass tolerance, 0.1 Da (the error tolerance); (iii) tryptic enzyme specificity with two missed cleavages allowed; (iv) monoisotopic precursor mass and fragment ion mass; (v) a fixed modification of cysteine carbamidomethylation; and (vi) variable modifications including iTRAQ (for cell protein quantitation only), lysine acetylation, deamidation on asparagine and glutamine, oxidation of methionine, and conversion of glutamic acid and glutamine to pyroglutamate.

For iTRAQ quantification, the peptide (with 99% confidence) for quantification was selected by PEAK Q module to calculate FDR and *P* value. The mass error tolerance was set to 0.1 Da, and the peptide score threshold (−10lg⁡*P*) was set to that corresponding to 1% FDR. The results of differentially expressed proteins were validated sequentially by the following criteria: the proteins must contain at least two unique high-scoring peptides, the proteins have *P* < 0.05 and FDR ≤ 1%, and the fold change of proteins ≥1.5. To be less restrictive in the identification of proteins with altered relative abundance in treated cells with respect to the control (untreated) group, a protein was included in the analysis when it was confidently identified in at least two biological replicates. When data sets obtained in two different treatments were compared, a two-group *t*-test was performed, considered statistically significant when *P* < 0.05 [25].

Given the fact that there might be significant number of short peptides which cannot be labelled by iTRAQ reagent for cell supernatant samples, label-free quantification method supported by PEAKS Q of PEAKS Studio v7.0 was used. For each run, about 1.5 *μ*g of the protein was analysed via LC-MS/MS. Biological triplicate untreated and treated were used in tandem repeats for LC-MS/MS procedure as described above, and the relative concentrations of proteins were compared. The mass shift between different runs was set to 50 ppm, and 1.0 min was used for evaluating the retention time shift tolerance. Featured peptides including PTMs mentioned above, with FDR threshold 1%, were included in the quantitative analysis. The result was validated sequentially with the charge of featured peptides between 2 and 5, fold change ≥1, and detected in more than one sample of the triplicate, while the FDR of protein was set to ≤1%, and the number of unique peptides and fold change of each protein were set to ≥1 and ≥2, respectively; peptide ratio versus quality-score and ratio versus average-area (MS signal intensity) were set to recommended values of 8, respectively. The proteins presented were clustered using one minus Pearson correlation [26].

### 2.9. Pathway Analysis

The proteins determined to be differentially expressed were imported in Ingenuity Pathway Analysis (IPA; Qiagen, Redwood City, CA). In pathway analysis, activation/inhibition of signalling pathways was predicted based on the up/downregulation of key regulators in the different experimental condition for previously published targets of this regulator. Significance of the activation or inhibition of pathways predicted by the analysis was tested by the Fisher Exact test *P* value, considering only the predictions with significant *P* value of <0.05 and a regulation *z*-score of <−2 or >2 for inhibition and activation, respectively.

### 2.10. Statistical Analysis

Statistical analysis was performed by the two-tailed *t*-test. Survival rate comparison among different groups was performed by log rank test, by using Prism 6.0 (GraphPad Software, San Diego). Results are considered as significant if *P* value is less than 0.05.

## 3. Results

### 3.1. Caerin 1.1 and Caerin 1.9 Inhibit Proliferation of TC-1 Cells with Additive Effect and without Cytotoxicity to Normal Cells

**Figure 1:**
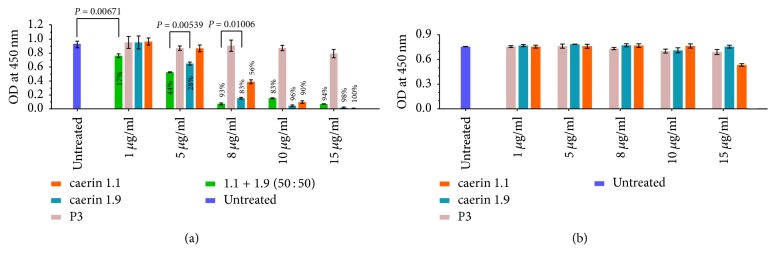
Caerin 1.1 and caerin 1.9 are able to inhibit the proliferation of TC-1 cells but not NP-69 cells, measured by MTT assay. (a) 1 × 10^5^ of TC-1 cells and (b) 1 × 10^5^ of NP-69 cells were cultured either in media alone or with different concentrations (15, 10, 8, 5, and 1 *μ*g/mL) of caerin 1.1, caerin 1.9, or P3 for 24 hours before MTT assay was performed. Inhibition magnitude (in percentage) in comparison with untreated cells was shown above or on the bars. Each bar represents the statistical mean from three biological replicates (performed in triplicate) and the error bars represent the standard deviation.

The antiproliferative activities of caerin 1.1 and caerin 1.9 against TC-1 cells were evaluated using MTT assays (Figure 1). Negative controls included (1) peptide (P3) with no antiproliferative effect and (2) cells without any peptide treatment [27]. With respect to the untreated control cells, at 15 *μ*g/mL, caerin 1.1, caerin 1.9, and their mixture showed an inhibition of 100%, 98%, and 94%, respectively (Figure 1(a)). Compared to P3, caerin 1.1 and caerin 1.9 significantly inhibited the proliferation of TC-1 cells at 15, 10, and 8 *μ*g/mL, respectively. The inhibition effect of caerin 1.1 or caerin 1.9 was concentration dependent as caerin 1.1 at 5 *μ*g/mL did not exhibit inhibition, whereas caerin 1.9 at 5 *μ*g/mL inhibited 28% of cell proliferation compared to untreated cells. At 1 *μ*g/mL, no antiproliferation effect was observed for either peptide. In addition, caerin 1.9 showed higher antiproliferative activity than that of caerin 1.1 at 10, 8, and 5 *μ*g/mL (Figure 1(a)). The additive effect of caerin 1.1 and caerin 1.9 (mass ratio 50 : 50) was also evaluated. At higher concentrations, including 15 and 10 *μ*g/mL, the mixture showed less activity; however there was a significant additive effect at lower concentrations of 8, 5, and 1 *μ*g/mL, compared to those of caerin 1.9 alone (*P* values = 0.01006 and 0.00539 at 8 and 5 *μ*g/mL, resp.). At 1 *μ*g/mL, the mixture only inhibited the proliferation of TC-1 cells, compared to untreated cells (*P* value = 0.00671).

To test whether caerin 1.1 or caerin 1.9 shows similar activities on noncancer cells, an MTT assay on NP69 cells was performed. Neither of the caerin peptides caused significant inhibition of transformed normal NP69 cell growth, with the exception of caerin 1.1 at a high concentration of 15 *μ*g/mL (Figure 1(b)). Similarly, caerin 1.1, caerin 1.9, and the mixture inhibit human cervical cancer cell line HeLa cell growth* in vitro* showing a dose dependence but do not inhibit the growth of another noncancer cell line HMC at the same concentration, respectively (Figure S1).

**Figure 2:**
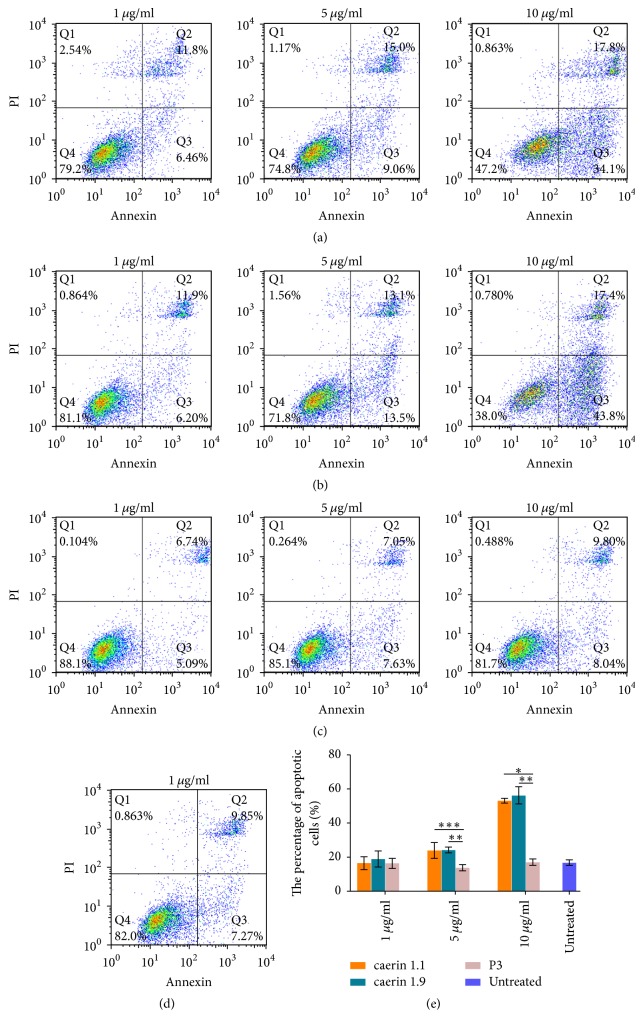
The cell apoptosis assay of TC-1 cells exposed to different concentrations of caerin 1.1, caerin 1.9, and P3 treatments and untreated TC-1 cells for 24 h by flow cytometric analysis after double-staining with Annexin/PI. (a), (b), and (c) show representative graphs obtained in the presence of caerin 1.1, caerin 1.9, and P3 at 10, 5, and 1 *μ*g/mL, respectively; (d) untreated TC-1 cells; (e) percentage of apoptotic (Q2 + Q3) and necrotic (Q1) cells detected in different treatments and control cells after 24 h. The bar graph represents the mean ± s.d. for *n* = 3 independent experiments for pooled early and late apoptotic cells. Statistical analysis was performed by the two-tailed* t*-test. ^*∗*^*P* < 0.001, ^*∗∗*^*P* < 0.01, and ^*∗∗∗*^*P* = 0.066 versus control.

The flow cytometric analysis confirmed that the antiproliferation effects of caerin 1.1 and caerin 1.9 occur as a result of peptide-induced TC-1 cell apoptosis (Figure 2). Caerin 1.1 (Figure 2(a)) and caerin 1.9 (Figure 2(b)), as with the control peptide P3, did not lead to TC-1 cell apoptosis at the low concentration (1 *μ*g/mL). However, they caused significantly more TC-1 cell apoptosis at 5 *μ*g/mL, compared to P3 and the untreated controls (Figure 2(d)). At 10 *μ*g/mL peptides, apoptosis rate increased to 40% and 43% for caerin 1.1 and caerin 1.9, respectively (Figure 2(e)), indicating that cell apoptosis induced by both caerin 1.1 and caerin 1.9 is dose-dependent. However, the mixture of caerin 1.1 and caerin 1.9 shows a similar apoptosis level compared to individual peptide (data not shown).

### 3.2. Quantitative Proteomic Analysis Reveals Differential Protein Expression in and outside TC-1 Cells following Caerin Peptide Treatments

**Figure 3:**
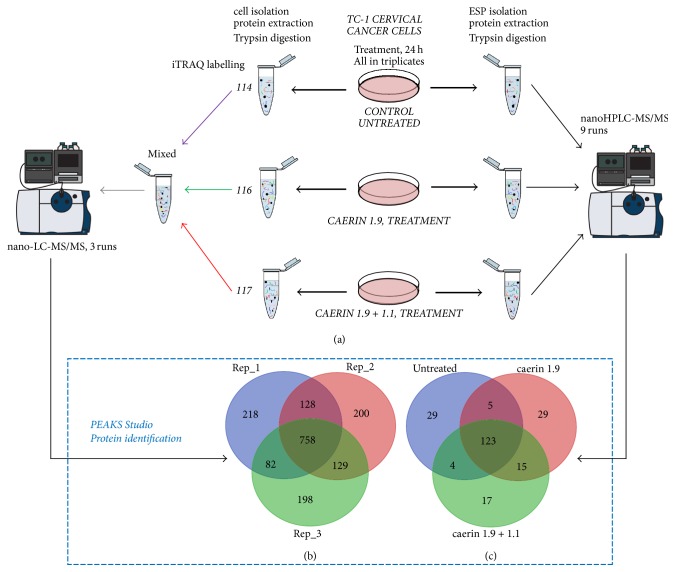
Overall workflow for characterisation of antiproliferation activity of peptide caerin 1.1 and caerin 1.9 against cervical cancer TC-1 cells, using bioassays in conjunction with quantitative proteomics. (a) Treated and untreated cell and supernatant proteins/peptides were extracted and labelled with iTRAQ, followed by identification and quantification with high-accuracy nano-LC Triple TOF-MS. (b) The comparison of proteins identified from cells (three replicates). (c) The comparison of protein identified from supernatant (three replicates used in untreated or treated conditions).

To gain molecular insights into the antiproliferative mechanisms of caerin 1.9 and caerin 1.9/1.1 (1 : 1) treatments to TC-1 cells, iTRAQ 4-plex labelling in conjunction with nano-LC-MS/MS was used to assess the differential expression of the proteome of TC-1 cells, while the ESP was quantitatively analysed using label-free quantitation method. A workflow for the preparation and analysis is shown in Figure 3(a). TC-1 cells were cultured with the addition of the single peptide and the mixture for 24 h. Each experiment was done in triplicate; TC-1 cells incubated only in cell culture media for the same time period were used as controls. In total, iTRAQ analysis was used in nine sets of experiments, that is, using 114 for controls, 116 for caerin 1.9 treated samples, and 117 for caerin 1.9/1.1 mixture treated samples (Figure 3(a)). Similar amounts of labelled proteins were mixed to generate three samples for further analysis. In terms of quantitative analysis of ESPs, starting from similar initial amount of whole proteins, label-free method based on featured peptides of proteins was used and nine samples were generated. All replicates were then subjected to nano-LC-MS/MS analysis.

Total cell protein profiles revealed a total of 1,713 proteins identified with an FDR < 1% (Figure 3(b)), with 758 proteins present in all replicates. After filtering and validation, an average of 779 proteins were quantified, including 353 proteins from at least two replicates (Table S1). For ESPs, 222 proteins were identified when all biological replicates were combined, among which 123 proteins were mutually identified in all replicates (Figure 3(c)); 90 proteins were quantified (see Table S2 for list of proteins identified and quantified). PCA was carried out to determine and compare the overall relationship among all samples (Figure S2). The combination of first and the second principal component captured more than 80% of the data variance. Triplicates with the caerin 1.9 treatment appeared to be more scattered, but they were closer to those treated by the mixture than to the controls.

To investigate protein expression changes and the underlying molecular pathways in response to TC-1 cell peptide treatments, the proteomic profiles at 24 h after treatment were compared. Those cell proteins and ESPs with fold change greater than 1.5 are shown in Figures S3–S6. Figures S3–S5 display the quantitative results of cell proteins in each replicate and Figure S6 shows the ESP quantitation with triplicate samples grouped together (see Tables S1 and S2 for the protein annotations).

Datasets of cell proteins obtained following treatment with caerin 1.9 and caerin 1.9/1.1 showed similarity, yet significant differences can also be observed. Among those proteins consistently quantifiable in all three replicates (with fold change ≥ 1.5 and FDR < 1%), the relative abundance of most proteins was upregulated after treatments. This was except for 9 proteins, including HNRNPCL1 (shared the same identified peptides with HNRNPCL2, HNRNPCL3, and HNRNPCL4), IPO5, CS, H2AFX (HIST2H2AB), ARF4, RPS14, TUBA1B (TUBA4A), HDLBP, and FLNA, which were downregulated with both treatments (Table S1). In addition, those proteins that showed statistically significant differences between caerin 1.9 and caerin 1.9/1.1 treatments included the TTR (three transthyretins were translated from this gene) and TP63 (tumour protein 63), which were found to be upregulated in caerin 1.9 treatments but downregulated in the mixture treatment (Table S1).

In terms of ESPs, the expression of 27 proteins was significantly suppressed after the two peptide treatments (Table S2), including the cytoplasmic HMGCLL1, NUP98, SMG7, PZP, AKAP9, EFCAB8, SUPT16H, RBP4, HBA1, WWC2, GC, HORMAD1, PCDHA9, SPTBN2, HEATR1, GTF3C2, APOE, C4B, EIF5B, PRRC2C, ABCF1, PCDHB7, PPFIA3, AMBP, FARSB, PLG and FBLN1. The proteins upregulated by both treatments, included LTF, THBS1, ZBTB4, TEX13D, OR5A2, SERPINC1, HIST2H2BF, PTPRD, GAS2L1, OR5H2, and CHGB. In addition, consistent upregulation of eleven proteins (AFP, SGK1, NEB, WDFY4, LUM, ALB, FHOD1, POTEE, RPL4, MT1E, and TF) was observed following treatment with caerin 1.9 alone. Three proteins (MYH14, TCTN1, and C5) were overexpressed following the mixture treatment. Four proteins (TMEM208, CCDC105, C9, and MROH2B) were upregulated consistently in all three replicates treated with the mixture, yet upregulated in only one sample after the caerin 1.9 treatments (Figure S5). The identification of the membrane proteins OR5A2, OR5H2, and TMEM208 with relatively high content may be explained by peptide-mediated membrane breakage.

### 3.3. Caerin 1.9 Treatment Modulates Biological Pathways in TC-1 Cells

**Figure 4:**
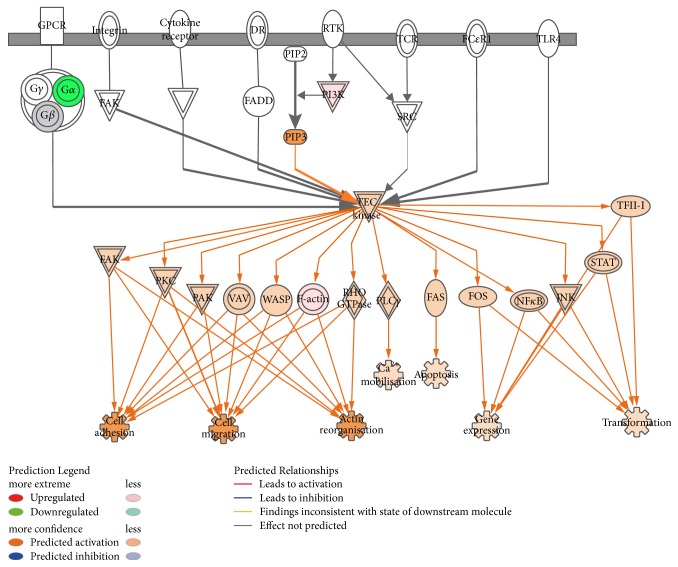
IPA identified “Tec kinase family signalling” as the canonical pathway in the cells with the highest predicted potential/significance of being affected by the treatment of caerin 1.9, based on iTRAQ data. The up/downregulations of proteins induced by caerin 1.9 are indicated by nodes with range of red and green intensities. The activation or inhibition of certain signalling was shown in orange or blue, respectively. Lines connecting the proteins represent known interactions, and arrows indicate the directions of up/downstream regulations.

Our quantitative protein analysis identified proteins with a fold change greater than 1.5 following caerin 1.9 treatment, and a FDR of 1% was used to determine statistically significant proteins differentially regulated between treatments. Only those proteins overlapping across at least two replicates were retained. These proteins were then subjected to Ingenuity Pathway Analysis (IPA) to help characterise potential biological pathways that were enhanced or suppressed in response to treatment. Pathway analysis showed that the downstream pathways of Tec kinase signalling (Figure 4) could be activated in caerin 1.9-treated cells. This includes PIP3 [phosphatidylinositol (3,4,5)-trisphosphate] induced activation of the Tec kinase signalling pathway in TC-1 cells, which then leads to the activation of all the downstream regulators including FAK, PKC, PAK, VAV, WASP, FAS, and so forth. Consequently, these regulators would affect several cellular processes in cells, including cell adhesion and migration, action reorganisation, Ca^2+^ mobilisation, apoptosis, gene expression, and transportation [28, 29].

The upregulation of PIP3 also contributed to the upregulation of ILK (integrin-linked kinase) in the ILK signalling pathway. This resulted in the activation of downstream regulators that control cell proliferation and tissue invasion and potentially cell motility and adhesion, opsonisation, and cytoskeletal reorganisation, as well as contributing to cell retraction, migration, and survival [17–19].

Another pathway that was significantly modulated following peptide treatment is the CXCR-4 signalling pathway (File S1), in which gene expression within the nucleus was suppressed, induced by the inhibition of ELK1 and EGR1. Upstream regulators found to be inhibited in the cytoplasm were ERK1/2, MEK1/2, c-RAF, RAS, SRC, FAK, and LNY. Though less significant, a number of the regulator proteins playing roles in cardiac hypertrophy signalling appeared to be activated, as shown in File S1, possibly associated with higher hypertrophic response. The dataset also suggested that the LXR/RXR pathway, a pathway related to the cytoplasm of hepatocytes, was suppressed significantly, and regulators inhibited included cholesterol metabolism, transport and efflux, and lipogenesis (File S1). An exception was that the biosynthesis of cholesterol could be promoted mainly as a result of the upregulation of FDFT-1 [30]. Several node proteins involving the interactions among eNOS, CaM, HSP90, AKT, and CAV1 in the NO signalling were upregulated, thus activating the entire eNOS-caveolin regulatory cycle; this has important influences on NO-dependent signalling in the vascular wall [31].

It is interesting to find that the VEGF signalling was potentially enhanced, as the interaction between VEGF and its receptor was more activated; as a consequence, closely associated nodes, including PXN, VCL, FAK, and *α*-actinin, were activated in the cytoplasm, resulting in active cell migration. BCL-2/XL and 14-3-3*σ*/FKHR appeared to function reversely in the modulation of cell survival. In contrast, lymphangiogenesis and angiogenesis were predicted to be suppressed resulting from the downregulation of nitric oxide (File S1).

Protein synthesis and translation were enhanced as a result of the p70S6K signalling pathway and more specifically were induced by activation of S6 and TAU factors. The upregulation of PKC in the thrombin signalling pathway could contribute to platelet aggregation within the nucleus. Other pathways identified with significant changes for treated TC-1 included cell integrin, leucocyte, and Rho family GTPase signalling (File S1).

IPA of the ESPs indicated that there were two signalling pathways that showed significant regulatory changes after caerin 1.9 treatment, including the supernatant acute phase signalling and LXR/RXR activation of macrophage pathways. The quantitative analysis indicated the upregulation of glucocorticoid in the supernatant acute phase signalling pathway, whose interaction with GCR leads to the activation of A2M, functioning as a protease inhibitor and cytokine transporter [32]. In addition, significant activation of STAT3, NF-*κ*B, and NF-IL6 can also be seen after treatment. Conversely, TCF 1, TCF 3, and TCF 4 were inhibited within nucleus, which downregulates AMBP, APOA1/2, TTR, and AHSC accordingly.

**Figure 5:**
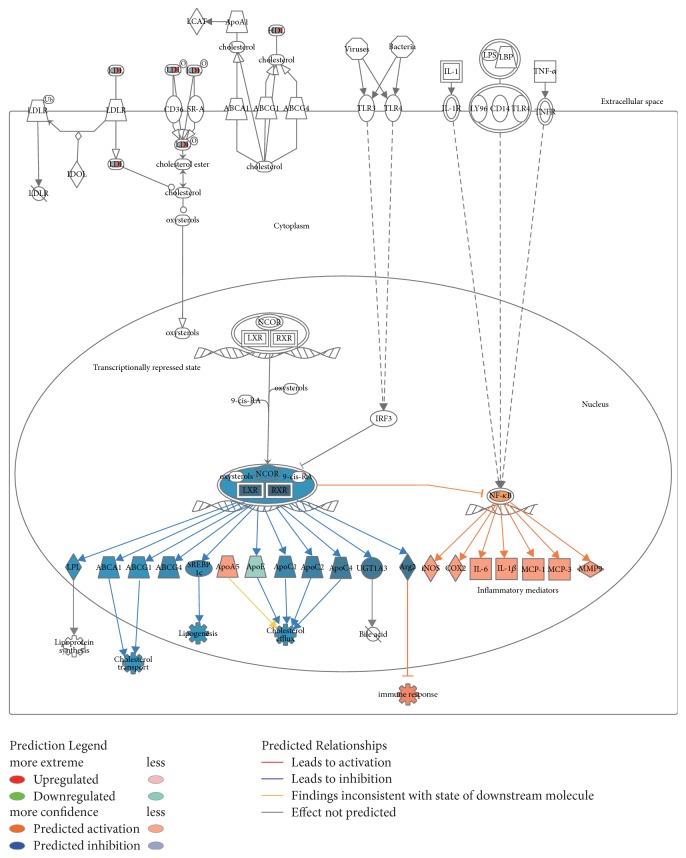
IPA identified “LXR/RXR activation” as the canonical pathway in the ESPs of TC-1 cells with the highest predicted potential/significance affected by the treatment with caerin 1.9, predicted based on label-free quantitation. The up/downregulations of proteins induced by caerin 1.9 are indicated by nodes with a range of red and green intensities. The activation or inhibition of certain signalling was shown in orange or blue, respectively. Lines connecting the proteins represent known interactions, and arrows indicate the directions of up/downstream regulations.

In the LXR/RXR pathway, the activation of NF-*κ*B could result in downstream factors becoming more active, including iNOS [33], COX-2 [34], IL-6/1*β* [35], MCP-1/3 [36], and MMP9 [37], all of which act as a network of inflammatory mediators. LXR, RXR, and NCOR were significantly inhibited, suggesting that the corresponding downstream regulators, which include LPL, ABCA-1, ABCG-1/4, SREBP-1c, APO-C1/2/4, UGT1A3, and Arg-2, were deactivated. The inhibition of these factors suppresses the cholesterol transport and efflux and lipogenesis but enhances immune response (Figure 5). Any change of the other two small molecule regulators, oxysterols and 9-cis-RA, could not be determined from this study.

### 3.4. Biological Implications

Caerins and a number of other peptides isolated from Australian tree frogs have been shown to exert antimicrobial, antifungal, and antiviral host-defence responses against different organisms at concentrations lower than those typically used in clinical reagents, and the effects of these peptides are cell-type- and dose-dependent [21, 22]. Caerin 1.1 inhibits the proliferation of several cancer cells by lysing the cells, which is a consequence of the peptide structural characteristic of helix-hinge-helix when approaching the cell membrane [38]. Before this study, the antiproliferative effect of caerin peptides on TC-1 cervical cancer cells had not previously been studied. In the present study, we assess the effect of caerin peptides on cervical cancer TC-1 cells and report the resultant impact on TC-1 cell proteins through high-resolution quantitative proteomic profiling using nano-LC, high-resolution MS/MS, and iTRAQ labelling. The iTRAQ and label-free quantitation methods were used to examine relative changes in the protein abundance in TC-1 cervical cancer cells and its ESPs, respectively. The integration of isobaric tagging of peptides, such as TMT (tandem mass tag) [39] and iTRAQ [40], and shotgun proteomic methods enable the identification and quantification of peptides using MS/MS [41] and permit large scale parallel proteomic analysis of multiple samples [42]. High resolution and accuracy and high peptide identification rates have contributed to the quality of label-free quantification [43, 44], which has been used more widely [45, 46].

We found that both caerin 1.9 and caerin 1.1 can inhibit TC-1 cell proliferation* in vitro* and, when applied together, have an additive effect. Importantly, at similar doses, these peptides did not inhibit the proliferation of the normal epithelial transformed cells, NP-69, except caerin 1.1 at a high concentration of 15 *μ*g/mL (Figure 1(b)). We also found that these two peptides are able to inhibit the proliferation of two human breast cancer cell lines, and the inhibition is also dosage dependent (data not shown). Moreover, the two peptides inhibit TC-1 tumour cell growth in a mouse model (data not shown). This suggests that the caerin antiproliferation ability might be associated with the interaction with the cancer cells' abnormal cell membrane. Caerin effects were supported by quantitative proteomic analysis, showing that proteins including HNRNPCL1 (shared the same identified peptides with HNRNPCL2, HNRNPCL3, and HNRNPCL4), IPO5, CS, H2AFX (HIST2H2AB), ARF4, RPS14, TUBA1B (TUBA4A), HDLBP, and FLNA were downregulated. The downexpression of TUBA1B, FLNA, and HNRNPCL1/2/3 indicated that the membrane integrity was negatively affected by the treatments, as these proteins are highly membrane associated, which thus reduced cell migration and proliferation. The CS protein is nuclear encoded and transported into the mitochondrial matrix, which catalyses the synthesis of citrate from oxaloacetate and acetyl coenzyme A [47], which is a crucial indicator of the cell energetic state and does influence the activity or specificity of multiple enzymes [48]. IPO5 encoded an important transporter protein for nucleocytoplasmic transport, which is an energy-dependent process [49]. ARF 3 and ARF 4 play roles in vesicular trafficking and can activate phospholipase D [50–52] and other ARF family members are involved in cytokinesis [53], cell adhesion [54], and tumour cell invasion [55]. ARF4 has been reported to increases AP-1 promoter activity and induce breast cancer cell migration [56]. The normal functioning of HDLBP requires significant amounts of energy [57]. Thus, the inhibition of these energy-dependent enzymes and regulators is strongly indicative that the treatments interfered with the energy metabolism of TC-1 cells.

Relatively increased abundance of TTR (transthyretin) was found in the cells treated with caerin 1.9 compared to the control and the peptide mixture (at the lowest concentration). TTR is a carrier protein that exhibits downregulation in ovarian cancer [58] and several other diseases, such as liver disease [59], malnutrition [60], and acute inflammation [61]. Thus, it is conceivable that caerin 1.9 could enhance the transport executed by TTR.

TP63 has critical functions in the development of stratified epithelial tissues, such as epidermis, breast, and prostate, yet its role in tumorigenesis remains controversial [62, 63]. Previous publications show genetic variation of TP63 may influence susceptibility to lung adenocarcinoma [64] and cancer in the Han population [65]; substantial overexpression of TP63 was found in cervical cancer tissues [66]. The significant downregulation of TP63 caused by the caerin mixture could contribute to their additive effect.

The rapid proliferation of tumour cells can result in accelerated receptor recycling and increased excretion/secretion of a variety of molecules including matrix components, adhesion molecules, and growth factors [67, 68], which could be consistent with our identification of membrane proteins in ESP samples, for instance, OR5A2, OR5H2, and TMEM208. In addition, a higher concentration of membrane and transporter proteins, including NEB, fibril organization LUM, transporter ALB, cytoplasmic protein FHOD1, RPL4, and TF, were identified following caerin 1.9 treatment. This indicates that once attached to the TC-1 cell membrane, caerin 1.9 is able to enhance transport and membrane reorganisation. SGK1 is showing higher expression in caerin 1.9-treated samples, suggesting a stronger cellular stress response and higher regulation towards cell survival [69], while this was not observed for the peptide mixture, which might be overcome by the additive effect.

The pathway analysis based on the iTRAQ quantitation revealed that Tec kinase was activated. Tec kinases have been found to be critical regulators of the T cell receptor signalling required for phospholipase C–*γ* activation [70]. This pathway leads to the activation of specialised protein kinases, including mitogen-activated protein kinase (MAPK) and phosphorylate transcription factors, to translocate to the cell nucleus. The LXR/RXR pathway activated in ESP samples would cause the expression of proinflammatory cytokines: TNF*α*, IL-1*β*, IL-6, chemokines including monocyte chemoattractant protein 1 (MCP-1), macrophage inflammatory protein 3*α* (MIP-3*α*), and IL-8, and other inflammation-promoting enzymes (see Figure 5). Activation of these pathways is particularly important for therapeutic vaccination and immunotherapy. The apoptosis caused by the treatment with caerin 1.9 and caerin 1.1 leads to the secretion of TNF*α*, IL-1*β*, IL-6, and MCP-1. These cytokines and chemokines are proinflammatory and will promote immune cell trafficking to the tumour site, enhance antigen presentation by dendritic cells, and consequently enhance adaptive immune responses [71].

Furthermore, the modulated signalling pathways identified, including integrin, ILK, CXCR4, Rho family GTPase, and RhoGDI, showed activated cytoskeletal rearrangement, cell motility and adhesion, membrane linkage, and ruffling, which also implies that caerin 1.9 interacts with cell membranes. The activation of cell survival predicted in several signalling pathways (e.g., p70S6K, ILK, CXCR4, and VEGF) could be involved in the adaptive response mechanisms that TC-1 cells employed to cope with the stress induced by caerin 1.9. From the proteomic analysis, it appears that deaths of TC-1 cells due to the peptide treatment may directly relate to the interaction between peptides and tumour cell membrane, coupled with apoptosis of the TC-1 cells, as the apoptotic cells only accounted for 25% of the total TC-1 cells.

The TME is complex and consists of many cell types including endothelial cells and their precursors, smooth-muscle cells, fibroblasts, myofibroblasts, neutrophils, granulocytes (eosinophils and basophils), mast cells, T, B, and natural killer lymphocytes, and antigen presenting cells [72]. All the cells within the TME can participate in tumour progression via different biological pathways [73–76]. The discovery and development of molecules and approaches that are able to activate or suppress certain pathways to promote an immune response and apoptosis of tumour cells are guiding more promising therapeutical techniques [77–79]. Caerin 1.9 and its combination with caerin 1.1 not only inhibit tumour growth and lead to tumour cell apoptosis but also promote the tumour cell to secrete more proinflammatory molecules, such as MCP-1* in vitro*. MCP-1 is a chemokine with the ability to attract macrophages and subsequently T cells to the tumour site. Therefore, if combined with therapeutic vaccine, local administration of caerin 1.1 and caerin 1.9 may increase the efficacy of a therapeutic vaccine, which may provide a novel, efficient way for the better treatment of cancer.

## 4. Conclusions

In conclusion, caerin 1.9 inhibited the proliferation of TC-1 cells* in vitro* and had dose-dependent additive effects when using in combination with caerin 1.1. The expression of many proteins was significantly altered after TC-1 cells were treated with the caerin peptides. Our findings indicate a link between Tec kinase signalling and LXR/RXR pathways and acute inflammation and apoptosis in response to the caerin 1.9. It induced adaptive responses to alleviate cellular stress, as shown by proteomic changes for factors activated in p70S6K, ILK, CXCR4, and VEGF pathways. When the stress reaches a threshold, cell death occurs accompanied by release of acute inflammatory response cytokines and chemokines. These changes may improve the immune suppressive environment and improve the efficacy of a therapeutic vaccine. Therefore, future studies to investigate caerin peptides* in vivo*, by using tumour models, is warranted to discover if they cause tumour cell death and simultaneously provoke proinflammatory responses. Overall, these accumulative results indicate that caerin 1.9 and caerin 1.1 are able induce TC-1 cell death* in vitro *via a mechanism different from regular apoptosis, which may be serve to target tumours* in vivo* and improve the TME.

## Data Availability

Data is included in the Supplementary Materials.

## Supplementary Materials

Supplementary Materials Figure S1: caerin 1.1 and caerin 1.9 are able to inhibit the proliferation of HeLa cells but not HMC cells, measured by MTT assay. 1 × 10^5^ of HeLa cells or 1 × 10^5^ of HMC cells was cultured either in media, or plus different concentrations (25, 20, 15, 10, and 5 *μ*g/ml) of caerin 1.1, caerin 1.9, the mixture of 1.1 and 1.9 (mass ratio 1 : 1) or P3 for 24 hours before MTT assay was performed. (A) Caerin 1.1 treated HeLa cells, (B) caerin 1.9 treated HeLa cells, (C) the mixture treated HeLa cells, (D) P3 treated HeLa cells, (E) caerin 1.1 treated HMC cells, (F) caerin 1.9 treated HMC, (G) the mixture treated HMC cells, and (H) P3 treated HMC cells. Each bar represents the statistical mean from three biological replicates (performed in triplicate) and the error bars represent the standard deviation. Figure S2: probabilistic PCA is used to calculate principal components. *x* and *y*-axes show principal component 1 and principal component 2 that explain 68.8% and 19.5% of the total variance, respectively. *N* = 9 data points. Figure S3: heatmap of differentially expressed proteins in TC-1 cells identified from iTRAQ analysis of the first biological replicate treated with caerin 1.9 and the mixture (caerin 1.9 plus caerin 1.1 at a mass ratio of 1 : 1) at 24 h and untreated cells as control. The figure was generated using PEAKS studio. The decreased and increased proteins are indicated by range of green and red intensities, respectively. Figure S4: heatmap of differentially expressed proteins in TC-1 cells identified from iTRAQ analysis of the second biological replicate treated with caerin 1.9 and the mixture (caerin 1.9 plus caerin 1.1 at a mass ratio of 1 : 1) at 24 h and untreated cells as control. The figure was generated using PEAKS studio. The decreased and increased proteins are indicated by range of green and red intensities, respectively. Figure S5: heatmap of differentially expressed proteins in TC-1 cells identified from iTRAQ analysis of the third biological replicate treated with caerin 1.9 and the mixture (caerin 1.9 plus caerin 1.1 at a mass ratio of 1 : 1) at 24 h and untreated cells as control. The figure was generated using PEAKS studio. The decreased and increased proteins are indicated by range of green and red intensities, respectively. Figure S6: heatmap of differentially expressed proteins in the SEPs of three biological replicates treated with caerin 1.9 and the mixture (caerin 1.9 plus caerin 1.1 at a mass ratio of 1 : 1) at 24 h and untreated cells as control. Label-free quantification module of PEAKS studio was used to calculate the log 2 (ratio) values. The decreased and increased proteins are indicated by range of blue and red intensities, respectively. See Table S2 for details of protein identification and quantitation. Table S1: protein identification and quantitation results of three biological replicates of TC-1 cells treated by caerin 1.9 and the mixture (caerin 1.9 plus caerin 1.1 at a mass ratio of 1 : 1), compared to the control. For each replicate, there are protein identified, supporting peptides, iTRAQ quantified proteins, and de novo only peptides with average local confidence greater than 80%. Table S2: protein identification and quantitation results of three biological replicates of ESPs with the treatments of caerin 1.9 and the mixture (caerin 1.9 plus caerin 1.1 at a mass ratio of 1 : 1), compared to the control. It lists protein identified in control, treatment of caerin 1.9, and the mixture, as well as associated supporting peptides, quantified proteins, and de novo only peptides with average local confidence greater than 80%. File S1: other significant modulated canonical pathways identified from differentially expressed proteins in the cells or ESPs of TC-1 cells, with the treatment of caerin 1.9.
