# Supplementary material for: Comparative Proteomic Study of the Antiproliferative Activity of Frog Host-Defence Peptide Caerin 1.9 and Its Additive Effect with Caerin 1.1 on TC-1 Cells Transformed with HPV16 E6 and E7
Source: Biomed Res Int. 2018 May 13;2018:7382351. doi: 10.1155/2018/7382351 (PMC5971270; doi:10.1155/2018/7382351)

Other significantly modulated pathways predicted from caerin 1.9-treated TC-1 cell proteome

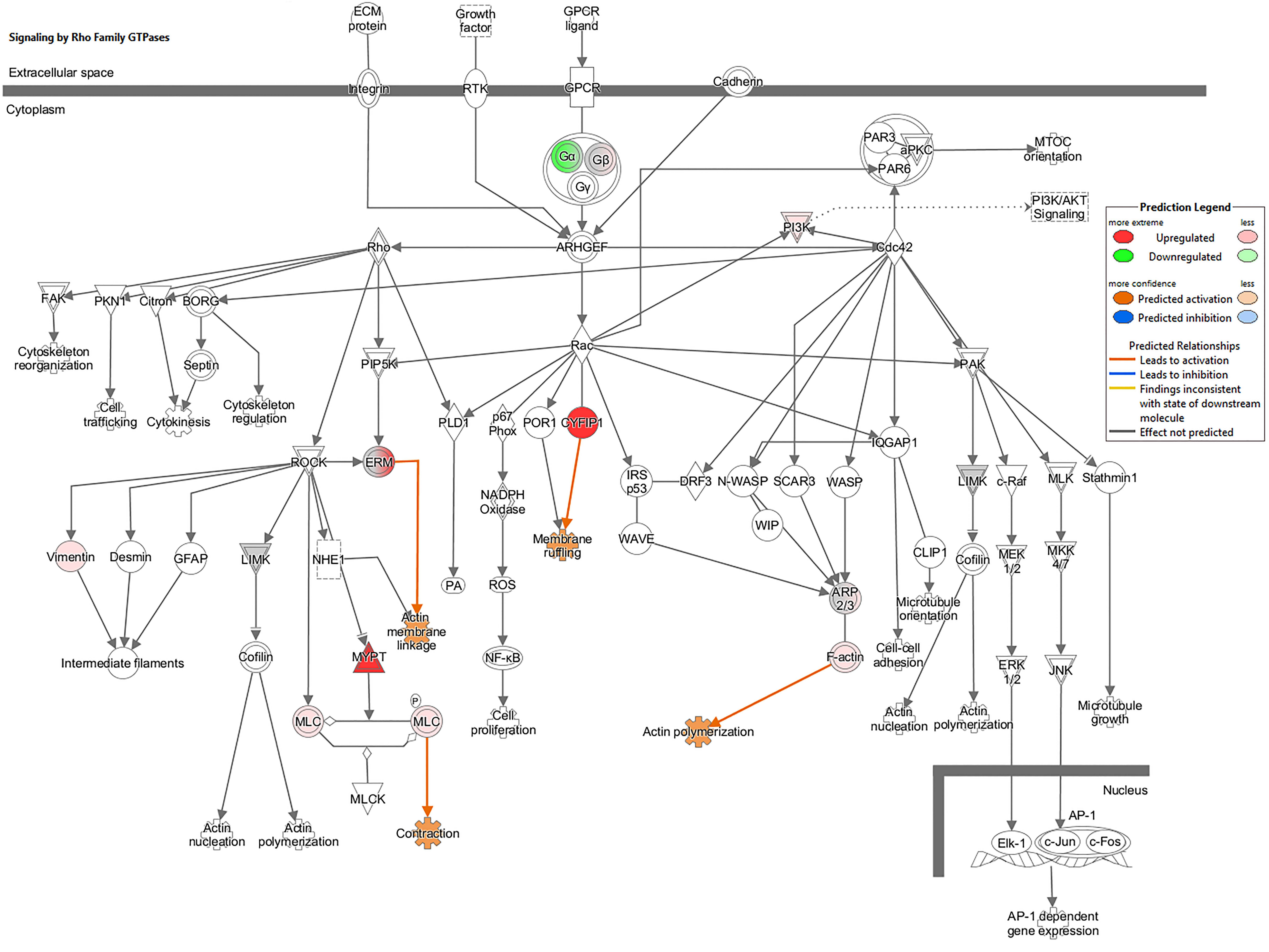

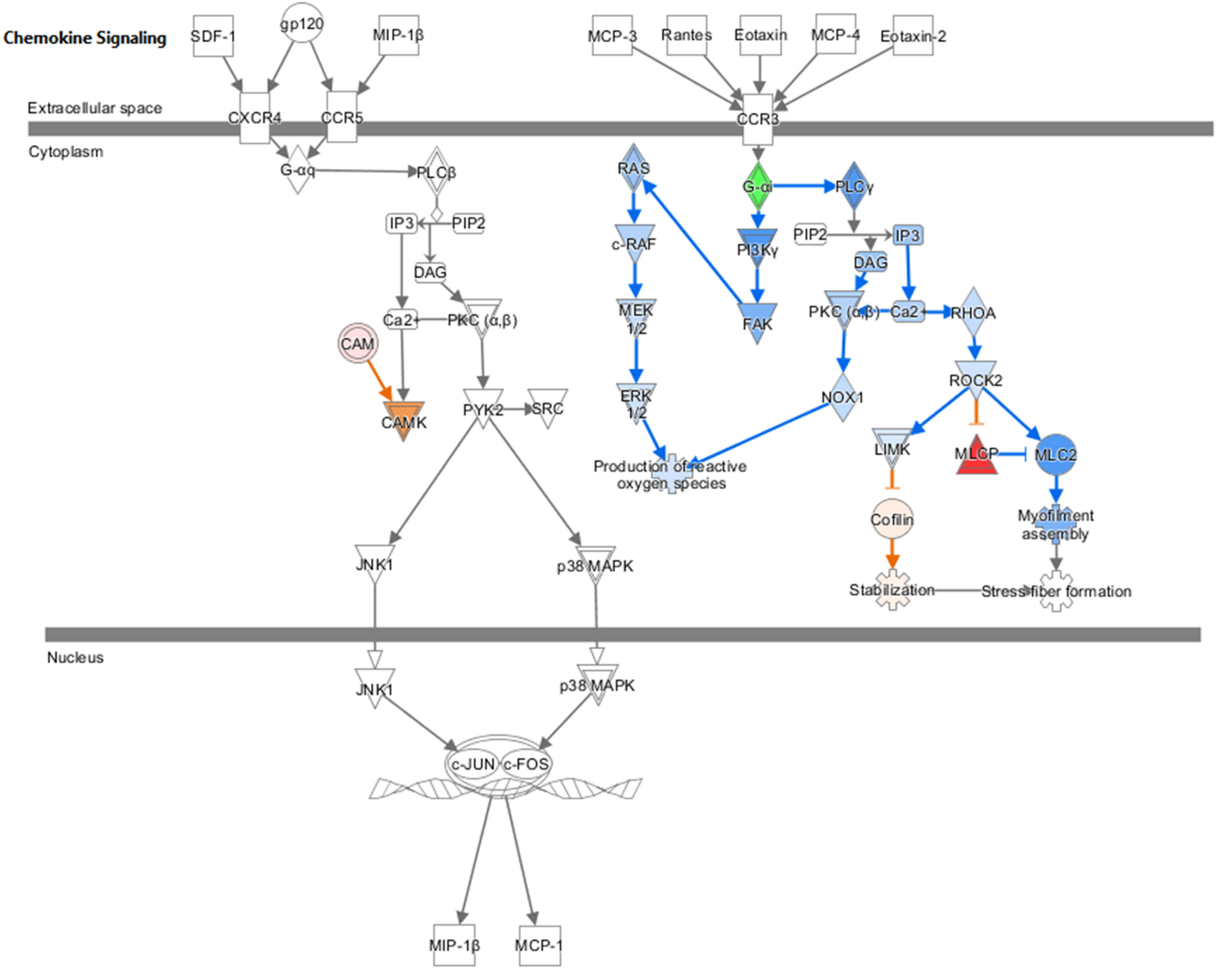
Chemokine Signaling
SDF-1
gp120
MIP-1β
MCP-3
Rantes
Eotaxin
MCP-4
Eotaxin-2
Extracellular space
CXCR4
CCR5
CCR3
Cytoplasm
G-αq
PLCβ
IP3
PIP2
DAG
Ca2
PKC (α,β)
CAM
CAMK
PYK2
SRC
RAS
c-RAF
MEK 1/2
ERK 1/2
G-αi
PI3Kγ
FAK
PLCγ
PIP2
IP3
DAG
PKC (α,β)
Ca2
RHOA
ROCK2
NOX1
Production of reactive oxygen species
LIMK
MLCP
MLC2
Cofilin
Myofilment assembly
Stabilization
Stress fiber formation
JNK1
p38 MAPK
Nucleus
JNK1
p38 MAPK
c-JUN
c-FOS
MIP-1β
MCP-1

CXCR4 Signalling

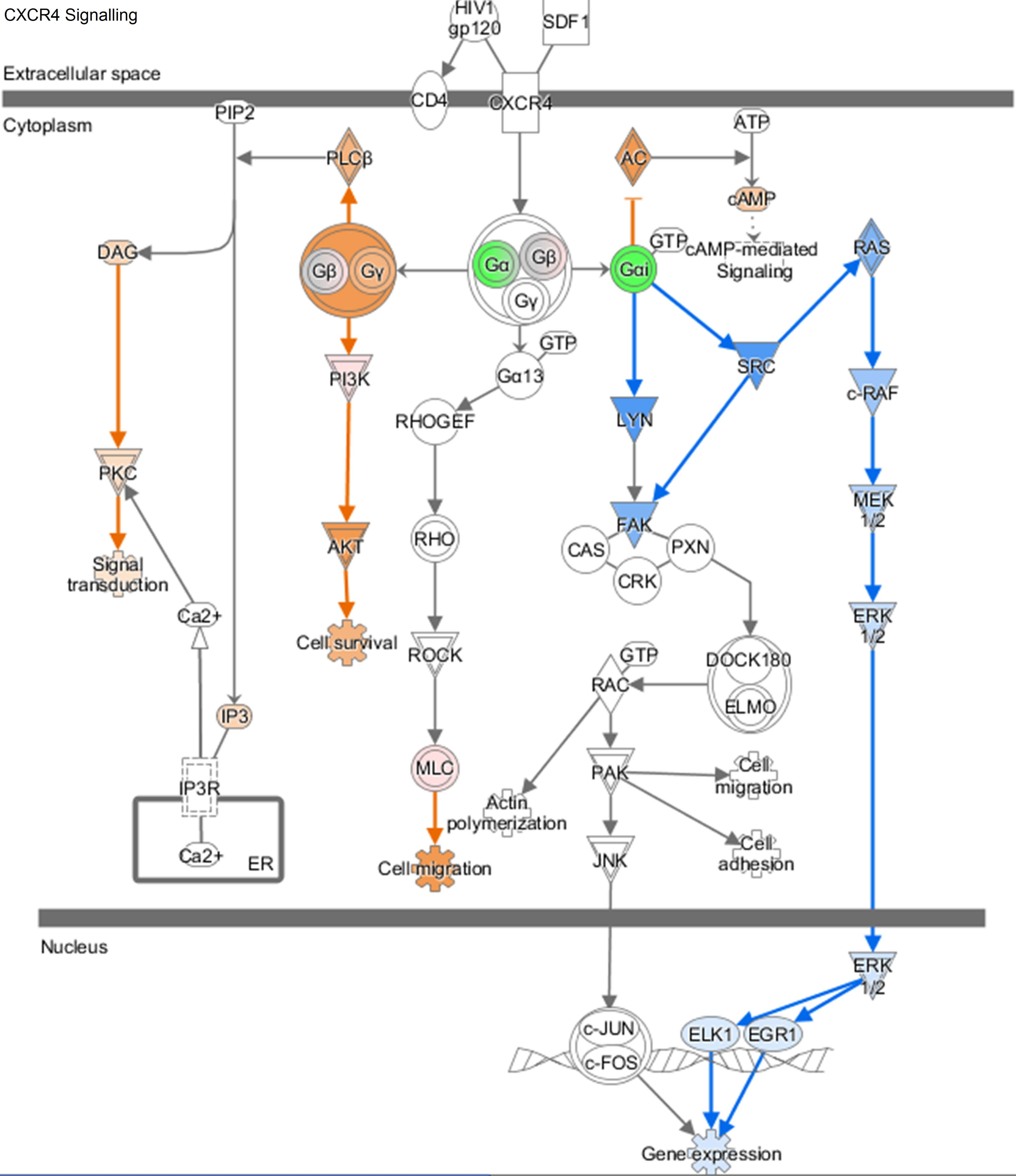

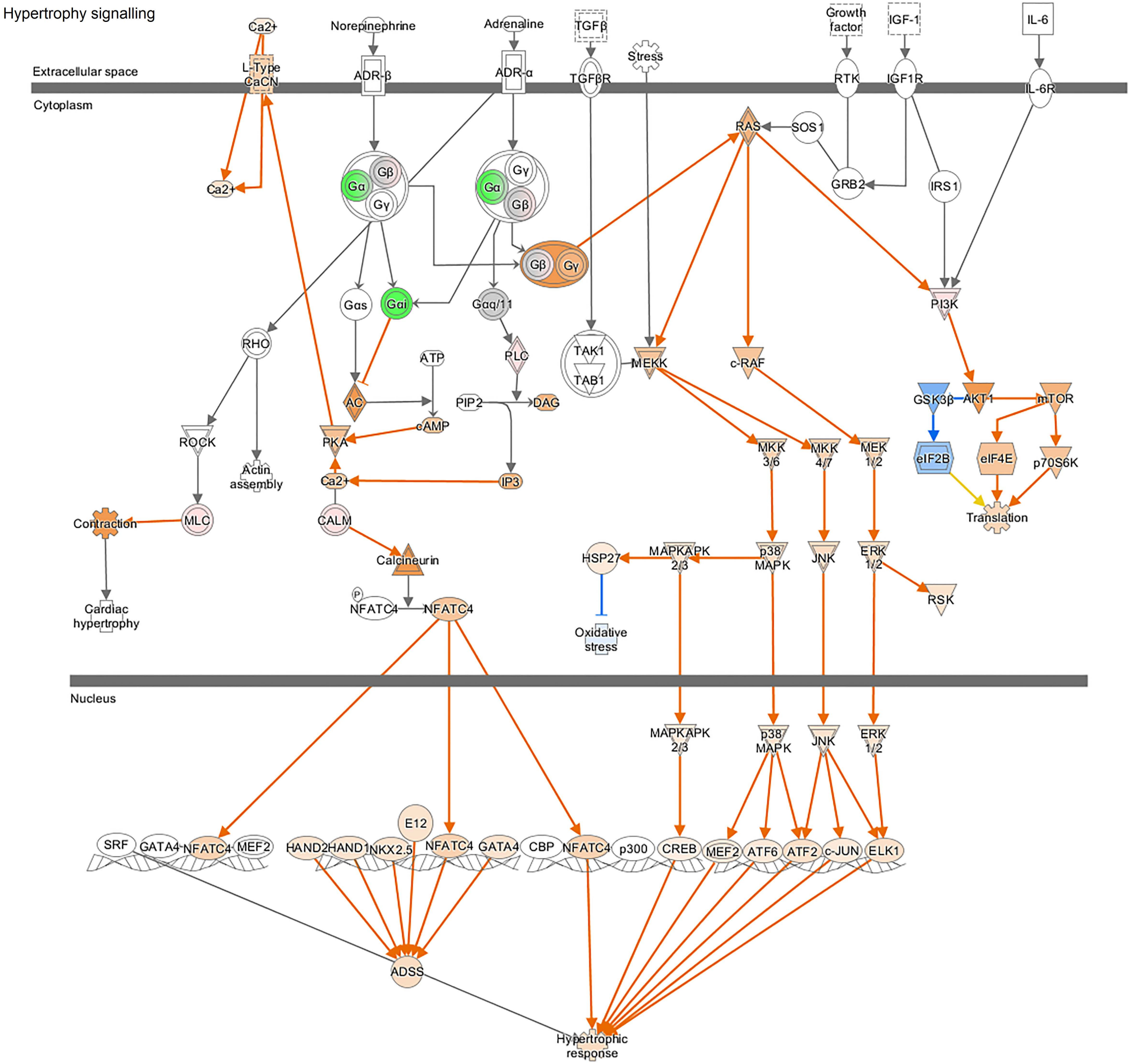

Hypertrophy signalling
Extracellular space
Cytoplasm
Nucleus
Ca2+
L-Type CaCN
Norepinephrine
ADR-β
Adrenaline
ADR-α
TGFβ
TGFβR
Stress
Growth factor
RTK
IGF-1
IGF1R
IL-6
IL-6R
RAS
SOS1
GRB2
IRS1
Gα
Gβ
Gγ
Gαs
Gαi
Gαq/11
RHO
ROCK
Actin assembly
MLC
Contraction
Cardiac hypertrophy
ATP
AC
cAMP
PKA
PLC
PIP2
DAG
IP3
CALM
Calcineurin
P
NFATC4
TAK1
TAB1
MEKK
c-RAF
MKK 3/6
MKK 4/7
MEK 1/2
p38 MAPK
JNK
ERK 1/2
RSK
MAPKAPK 2/3
HSP27
Oxidative stress
PI3K
GSK3β
AKT1
mTOR
eIF2B
eIF4E
p70S6K
Translation
SRF
GATA4
MEF2
HAND2
HAND1
NKX2.5
E12
CBP
p300
CREB
ATF6
ATF2
c-JUN
ELK1
ADSS
Hypertrophic response

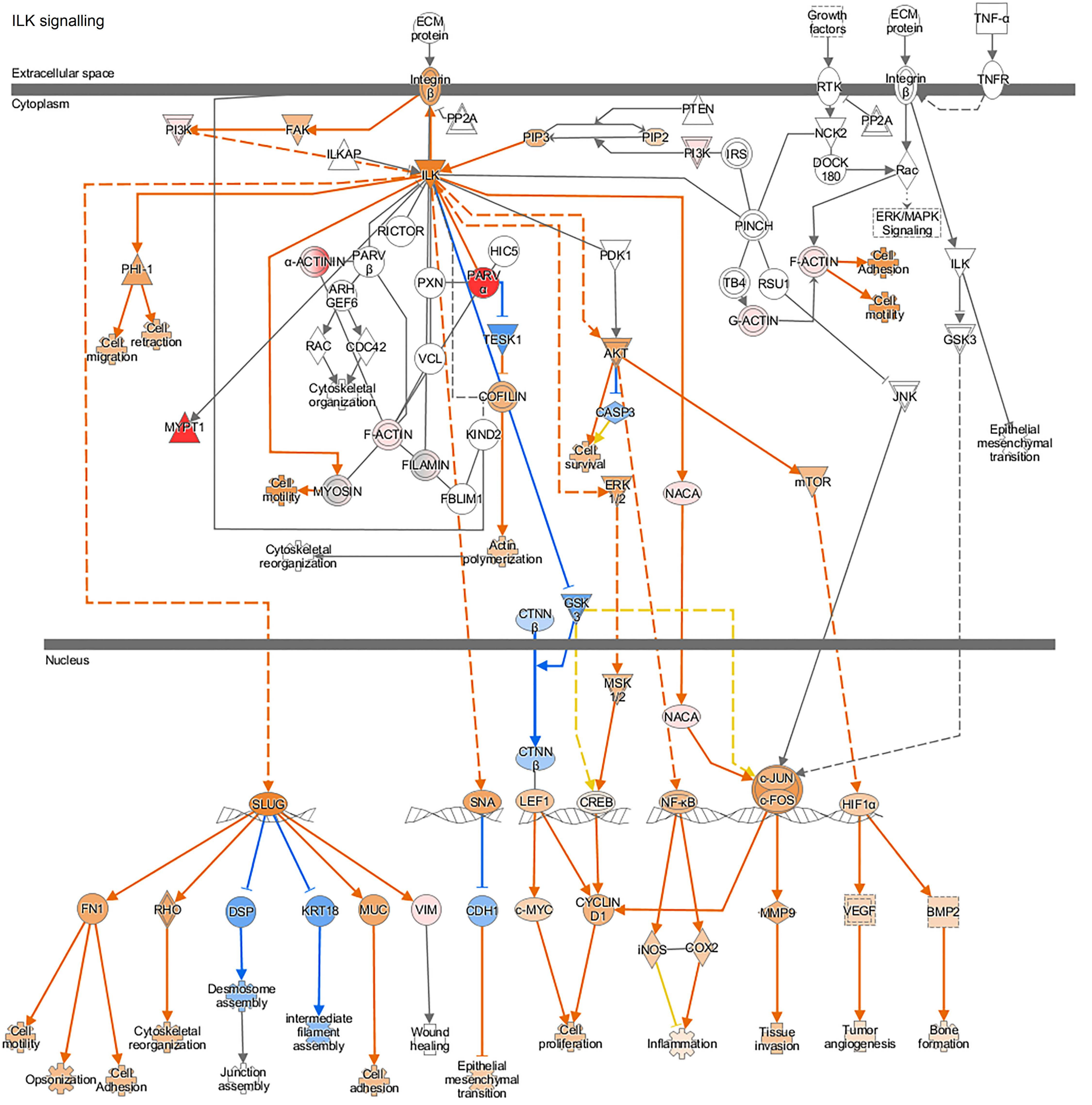
ILK signalling
Extracellular space
Cytoplasm
Nucleus
ECM protein
Integrin β
PP2A
PI3K
FAK
ILKAP
ILK
PIP3
PIP2
PTEN
IRS
Growth factors
RTK
NCK2
DOCK 180
Rac
ERK/MAPK Signaling
TNF-α
TNFR
PINCH
TB4
RSU1
G-ACTIN
F-ACTIN
Cell Adhesion
Cell motility
GSK3
JNK
Epithelial mesenchymal transition
RICTOR
HIC5
α-ACTININ
PARV β
ARH GEF6
PXN
PARV α
RAC
CDC42
Cytoskeletal organization
VCL
TESK1
COFILIN
KIND2
FILAMIN
MYOSIN
FBLIM1
PHI-1
Cell migration
Cell retraction
MYPT1
PDK1
AKT
CASP3
Cell survival
ERK 1/2
NACA
mTOR
Cytoskeletal reorganization
Actin polymerization
CTNN β
GSK 3
MSK 1/2
c-JUN
c-FOS
SLUG
SNA
LEF1
CREB
NF-κB
HIF1α
FN1
RHO
DSP
KRT18
MUC
VIM
CDH1
c-MYC
CYCLIN D1
iNOS
COX2
MMP9
VEGF
BMP2
Opsonization
Desmosome assembly
Junction assembly
intermediate filament assembly
Cell adhesion
Wound healing
Cell proliferation
Inflammation
Tissue invasion
Tumor angiogenesis
Bone formation

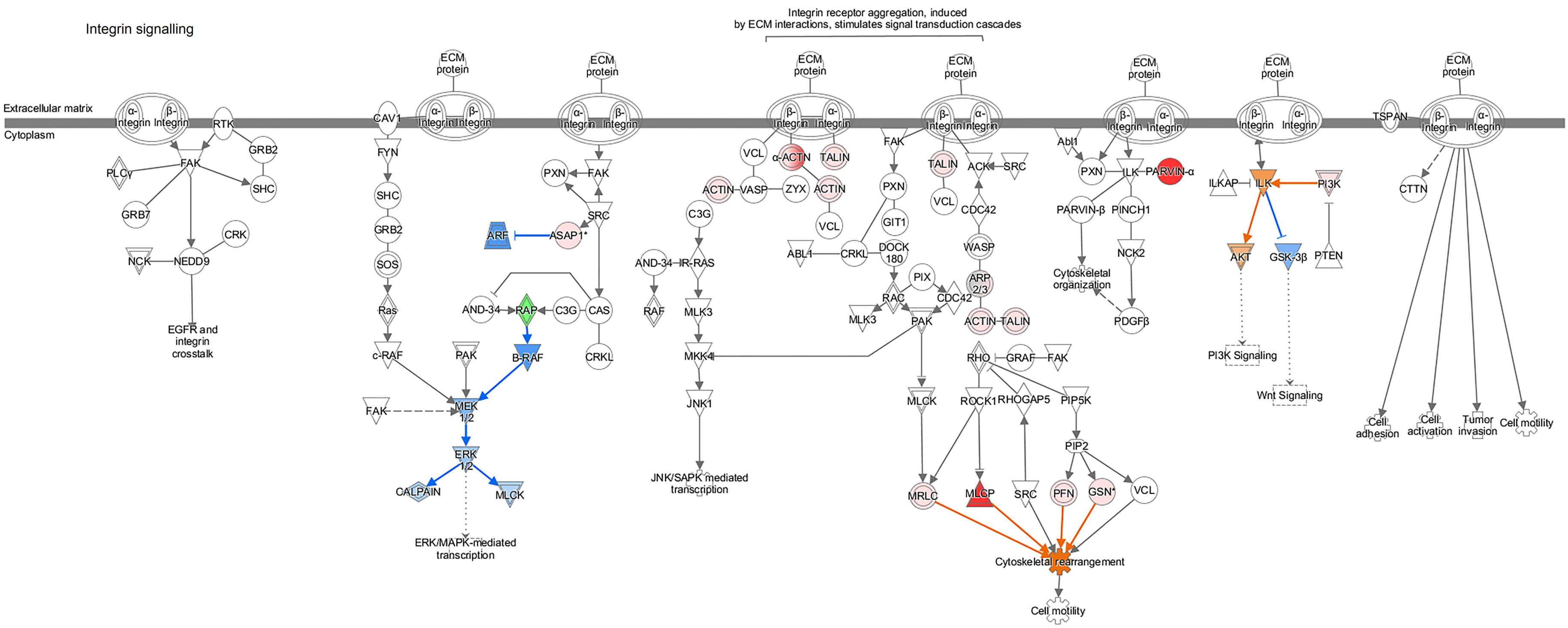
Integrin signalling
Integrin receptor aggregation, induced by ECM interactions, stimulates signal transduction cascades
Extracellular matrix
Cytoplasm
ECM protein
α-Integrin
β-Integrin
RTK
FAK
GRB2
PLCγ
SHC
GRB7
CRK
NCK
NEDD9
EGFR and integrin crosstalk
CAV1
FYN
SHC
GRB2
SOS
Ras
c-RAF
PAK
B-RAF
FAK
MEK 1/2
ERK 1/2
CALPAIN
MLCK
ERK/MAPK-mediated transcription
PXN
SRC
ARF
ASAP1*
AND-34
RAP
C3G
CAS
CRKL
VCL
α-ACTN
TALIN
ACTIN
VASP
ZYX
C3G
AND-34
R-RAS
RAF
MLK3
MKK4
JNK1
JNK/SAPK mediated transcription
PXN
GIT1
ABL1
CRKL
DOCK 180
PIX
RAC
CDC42
MLK3
PAK
MLCK
MRLC
ACK
CDC42
WASP
ARP 2/3
ACTIN
TALIN
RHO
GRAF
ROCK1
RHOGAP5
PIP5K
PIP2
MLCP
SRC
PFN
GSN*
VCL
Cytoskeletal rearrangement
Cell motility
Abl1
PXN
ILK
PARVIN-α
PARVIN-β
PINCH1
NCK2
Cytoskeletal organization
PDGFβ
ILKAP
ILK
PI3K
AKT
GSK-3β
PTEN
PI3K Signaling
Wnt Signaling
TSPAN
CTTN
Cell adhesion
Cell activation
Tumor invasion
Cell motility

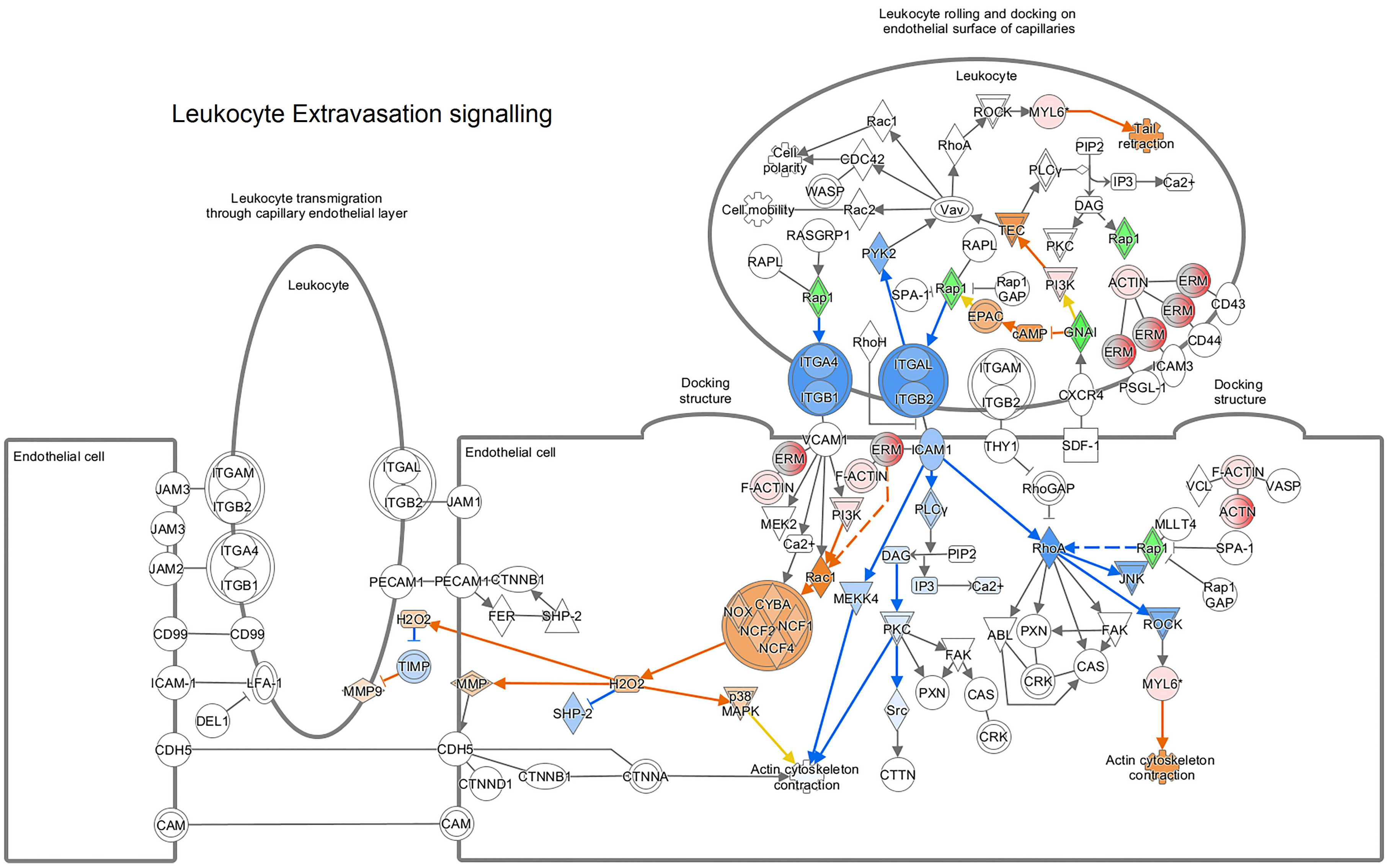
Leukocyte Extravasation signalling
Leukocyte rolling and docking on endothelial surface of capillaries
Leukocyte
Leukocyte transmigration through capillary endothelial layer
Leukocyte
Endothelial cell
Endothelial cell
Docking structure
Docking structure
ROCK
MYL6*
Tail retraction
Rac1
RhoA
PIP2
Cell polarity
CDC42
PLCγ
IP3
Ca2+
WASP
DAG
Cell mobility
Rac2
Vav
TEC
Rap1
RASGRP1
PKC
PYK2
RAPL
RAPL
PI3K
ACTIN
ERM
Rap1
SPA-1
Rap1
Rap1 GAP
CD43
ERM
EPAC
cAMP
GNAI
ERM
CD44
RhoH
ITGA4
ITGAL
ITGAM
ERM
ICAM3
ITGB1
ITGB2
ITGB2
CXCR4
PSGL-1
VCAM1
ERM
ICAM1
THY1
SDF-1
ERM
F-ACTIN
F-ACTIN
RhoGAP
VCL
F-ACTIN
VASP
F-ACTIN
PI3K
PLCγ
ACTN
MEK2
MLLT4
Ca2+
DAG
PIP2
RhoA
Rap1
SPA-1
Rac1
IP3
Ca2+
JNK
Rap1 GAP
NOX
CYBA
NCF2
NCF1
NCF4
MEKK4
PKC
ROCK
ABL
PXN
FAK
FAK
PXN
CAS
CAS
CRK
CRK
Src
MYL6*
CTTN
Actin cytoskeleton contraction
Actin cytoskeleton contraction
ITGAM
ITGAL
JAM3
ITGB2
ITGB2
JAM1
JAM3
ITGA4
JAM2
ITGB1
PECAM1
PECAM1
CTNNB1
FER
SHP-2
H2O2
CD99
CD99
TIMP
ICAM-1
LFA-1
MMP9
MMP
H2O2
p38 MAPK
SHP-2
DEL1
CDH5
CDH5
CTNND1
CTNNB1
CTNNA
CAM
CAM

LXR/RXR activation

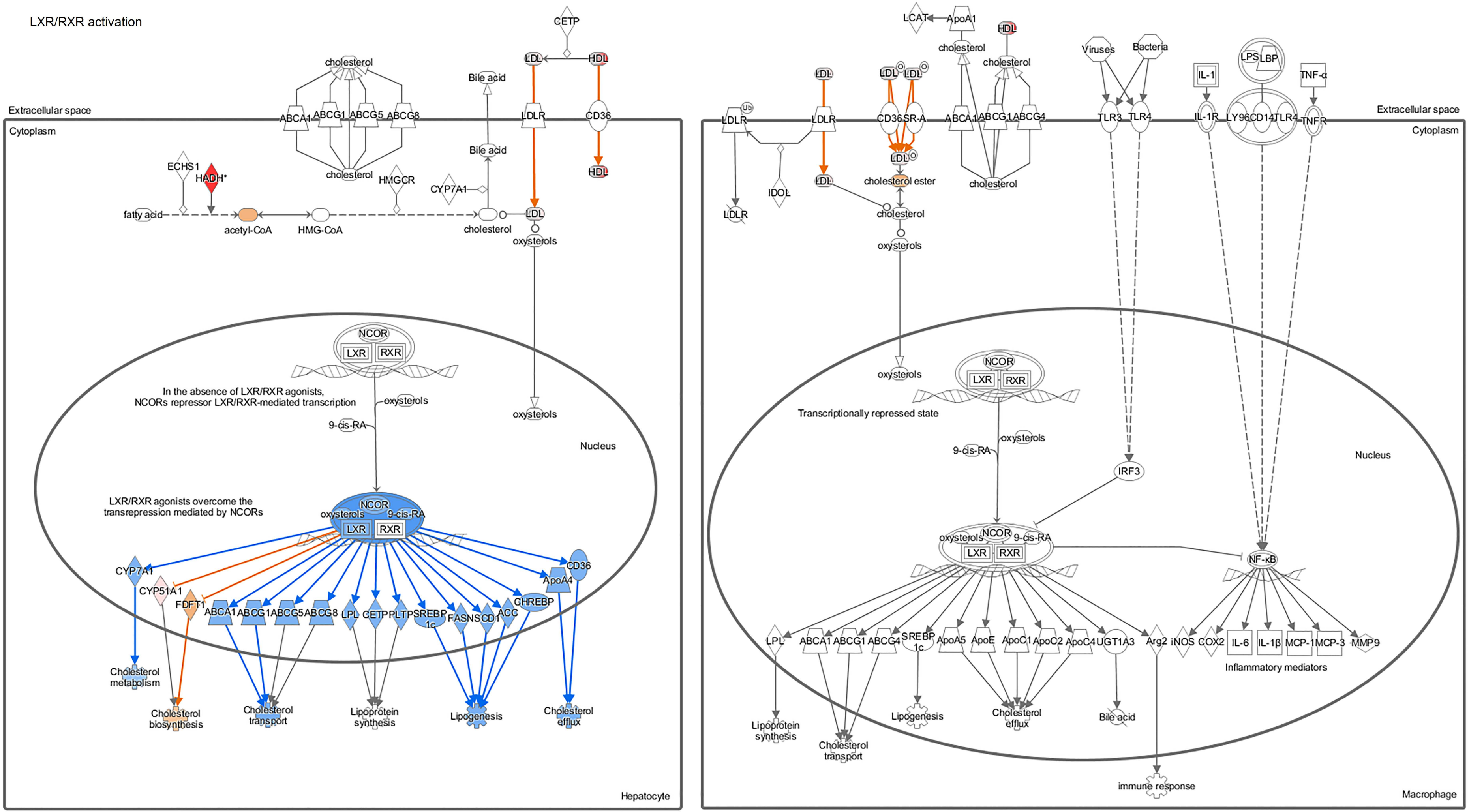

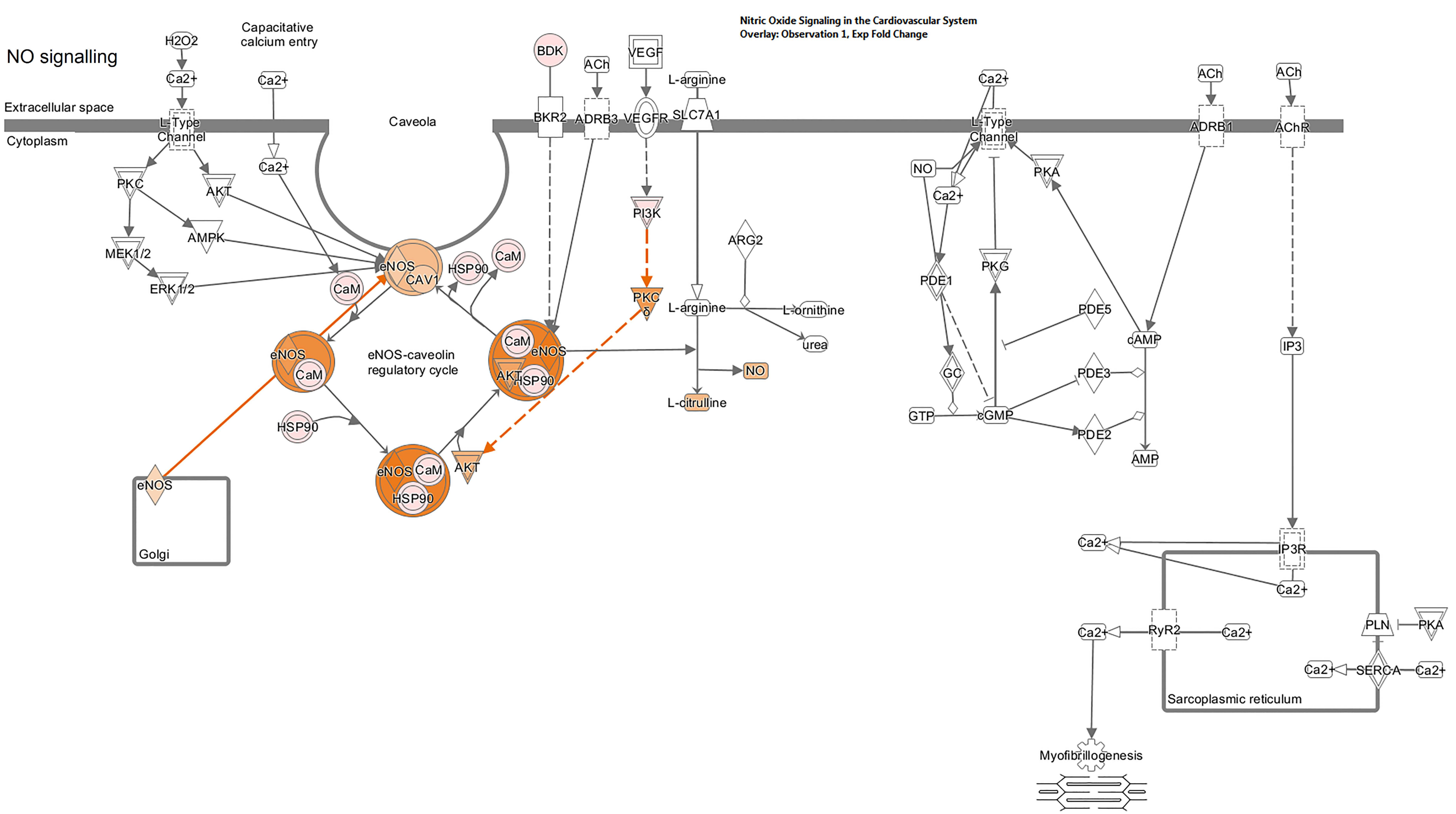
NO signalling
Nitric Oxide Signaling in the Cardiovascular System
Overlay: Observation 1, Exp Fold Change
Extracellular space
Cytoplasm
Capacitative calcium entry
Caveola
H2O2
Ca2+
L-Type Channel
PKC
AKT
AMPK
MEK1/2
ERK1/2
CaM
eNOS
CAV1
HSP90
BDK
BKR2
ACh
ADRB3
VEGF
VEGFR
L-arginine
SLC7A1
PI3K
PKC δ
ARG2
L-ornithine
urea
NO
L-citrulline
eNOS-caveolin regulatory cycle
Golgi
NO
PKA
PDE1
PKG
GC
GTP
cGMP
PDE5
PDE3
PDE2
cAMP
AMP
ADRB1
AChR
IP3
IP3R
RyR2
PLN
SERCA
Sarcoplasmic reticulum
Myofibrillogenesis

P70S6K signalling

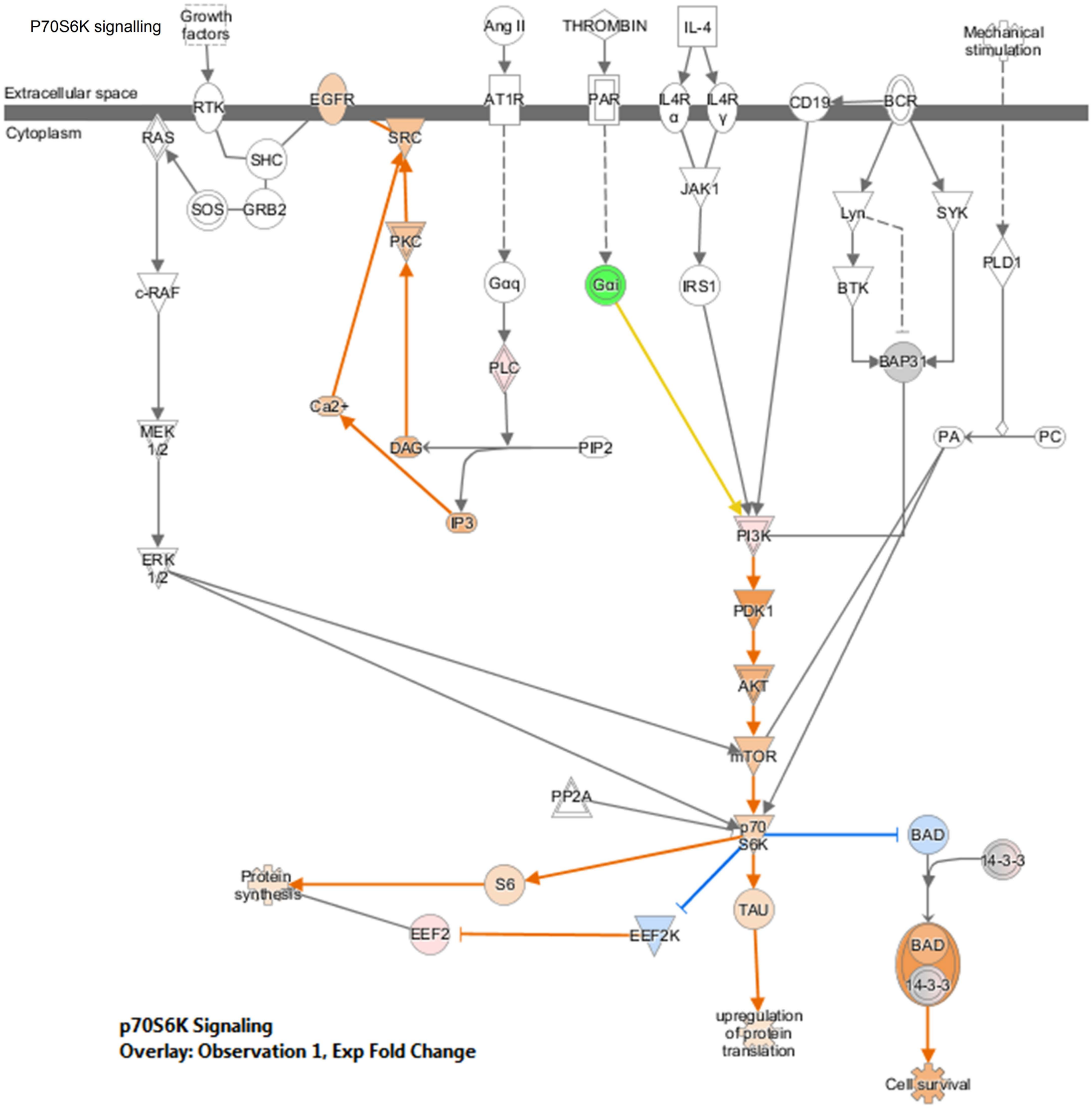

p70S6K Signaling
Overlay: Observation 1, Exp Fold Change

RhoGDI Signaling

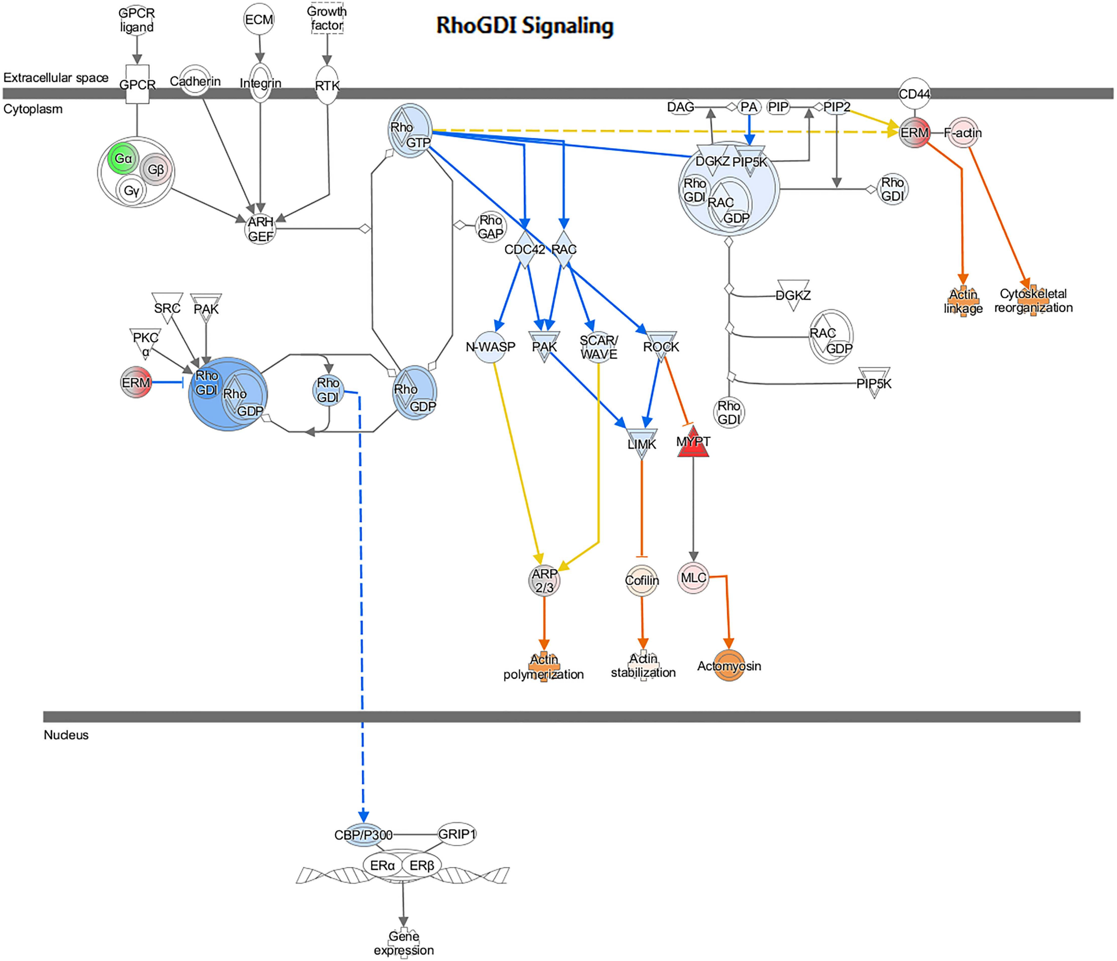

Thrombin signalling

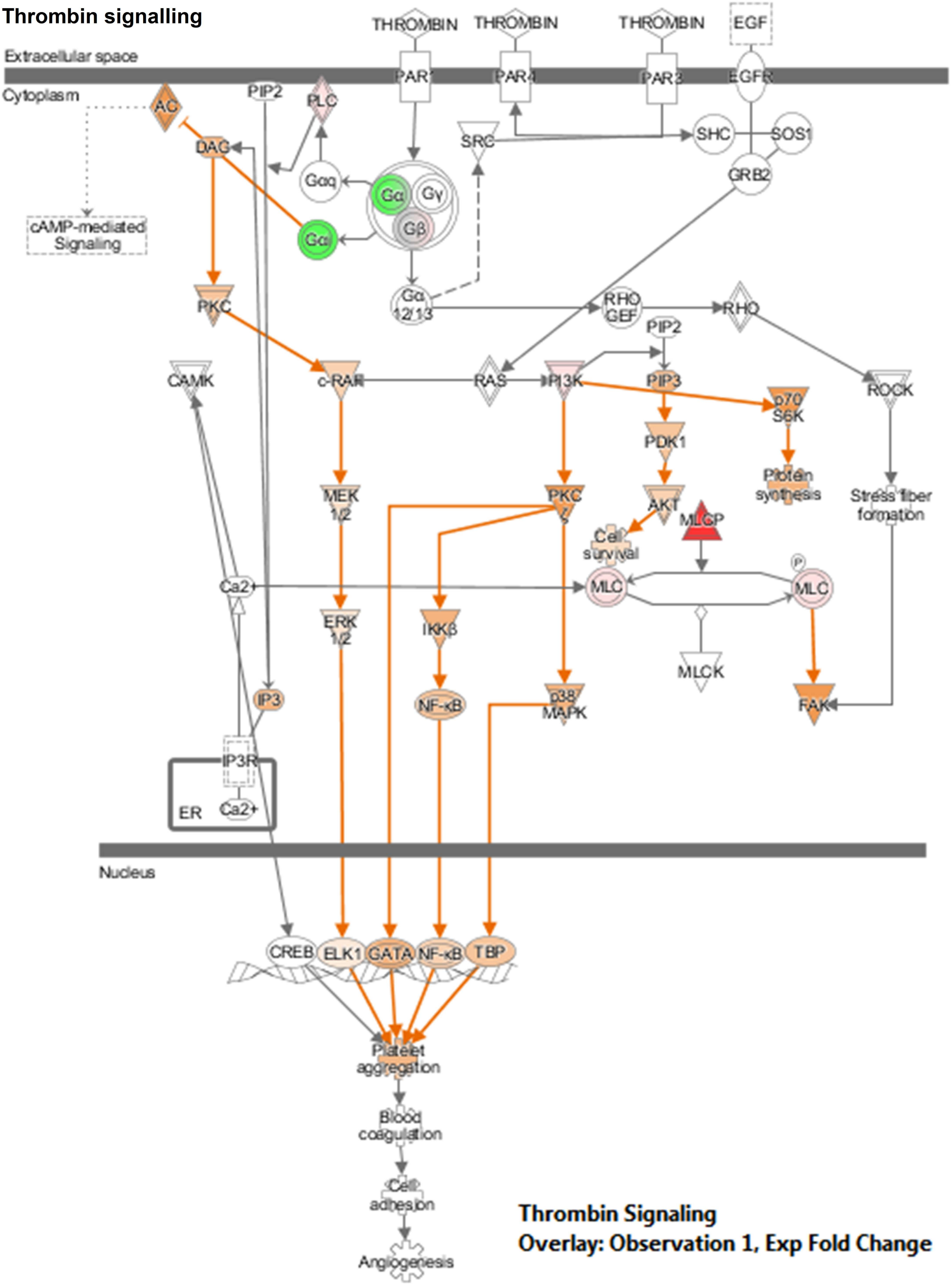

Thrombin Signaling
Overlay: Observation 1, Exp Fold Change

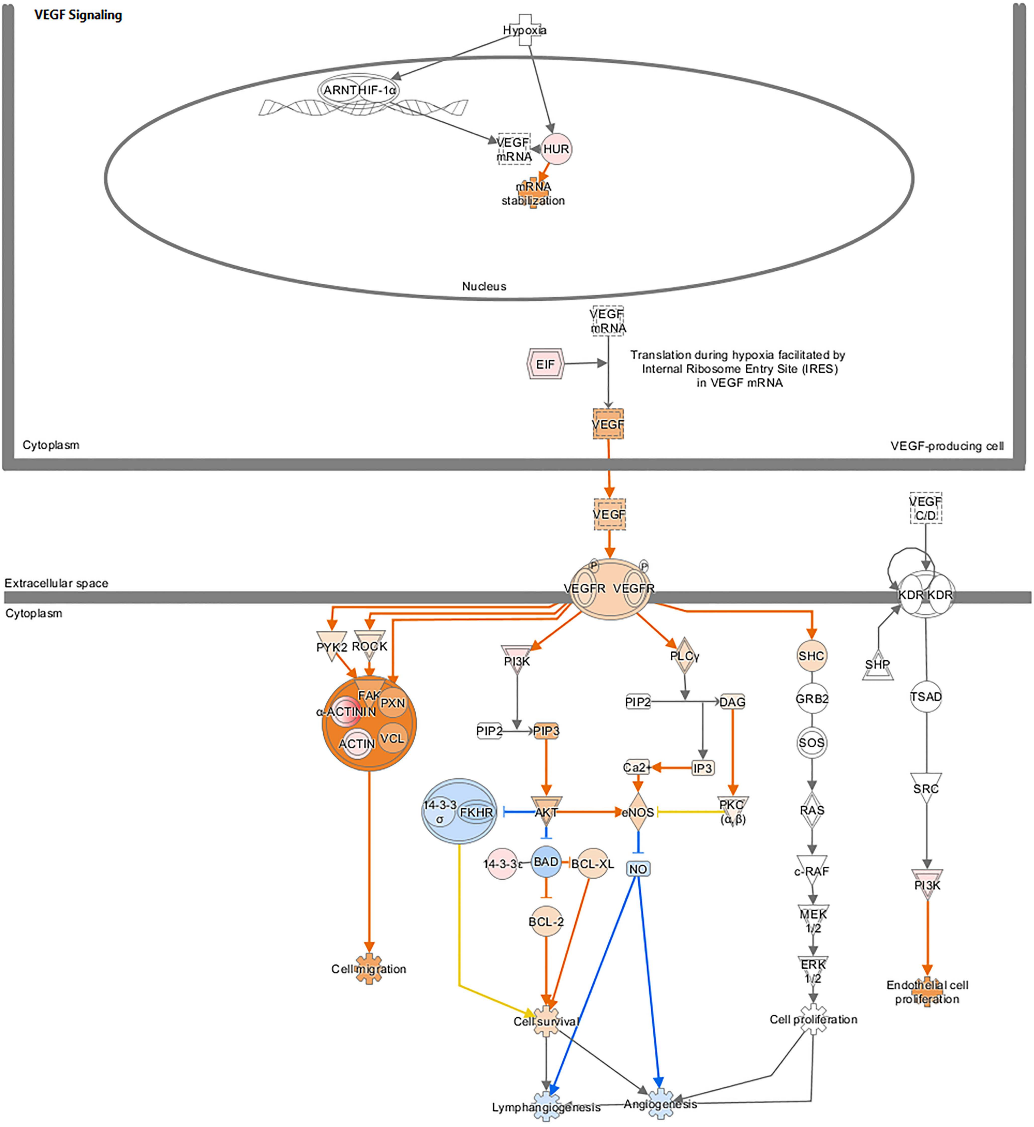
VEGF Signaling
Hypoxia
ARNT
HIF-1α
VEGF mRNA
HUR
mRNA stabilization
Nucleus
VEGF mRNA
EIF
Translation during hypoxia facilitated by Internal Ribosome Entry Site (IRES) in VEGF mRNA
VEGF
Cytoplasm
VEGF-producing cell
VEGF
VEGF C/D
VEGFR
VEGFR
KDR
KDR
Extracellular space
Cytoplasm
PYK2
ROCK
FAK
PXN
α-ACTININ
ACTIN
VCL
PI3K
PIP2
PIP3
PLCγ
PIP2
DAG
Ca2+
IP3
14-3-3 σ
FKHR
AKT
eNOS
PKC (α,β)
14-3-3ε
BAD
BCL-XL
NO
BCL-2
SHC
GRB2
SOS
RAS
c-RAF
MEK 1/2
ERK 1/2
SHP
TSAD
SRC
PI3K
Cell migration
Cell survival
Cell proliferation
Endothelial cell proliferation
Lymphangiogenesis
Angiogenesis

Supernatant acute phase signaling,
significantly modulated in ESPs, post caerin 1.9 treatment

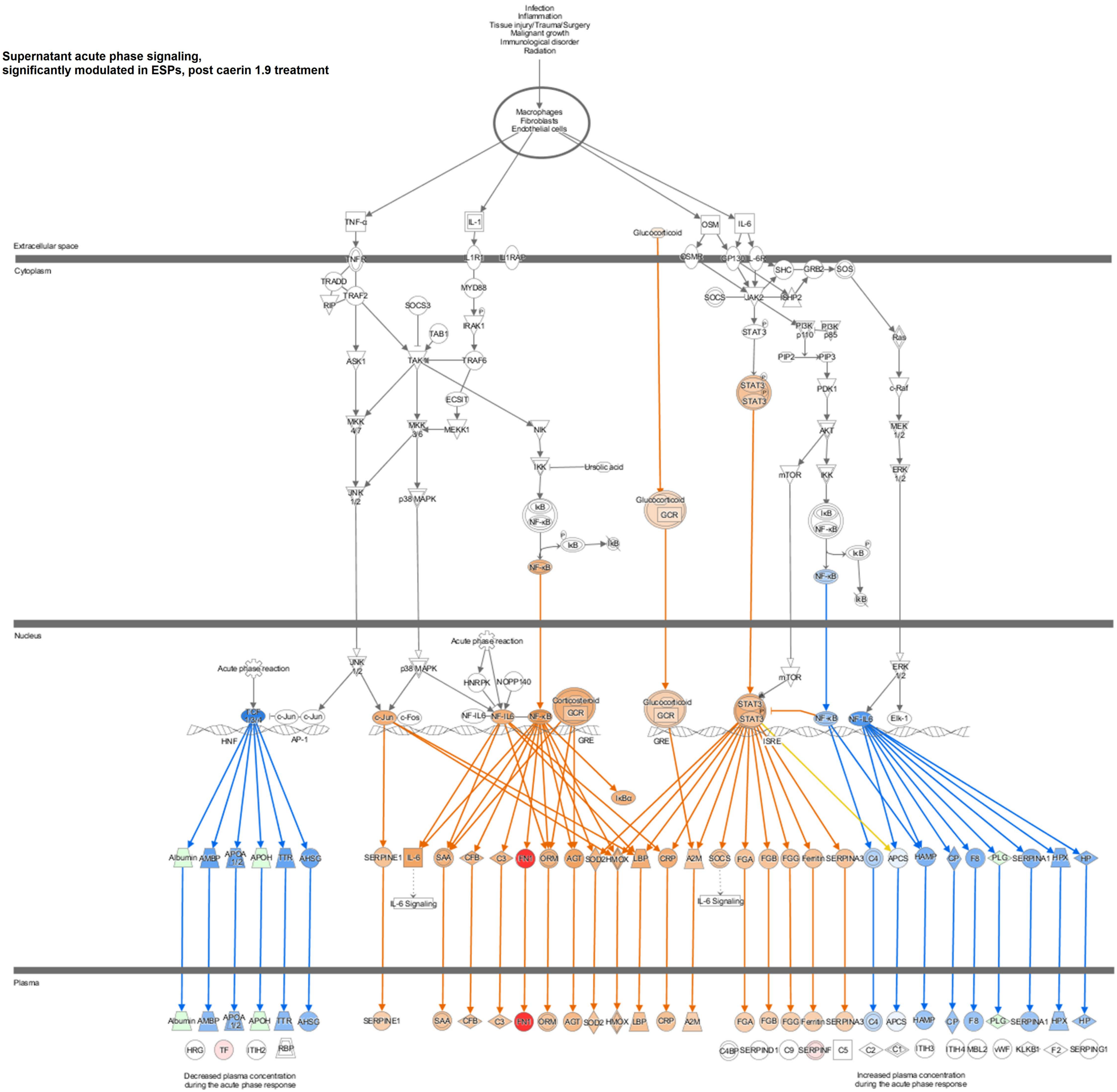

Supplement: Supplementary Materials — Figure S1: caerin 1.1 and caerin 1.9 are able to inhibit the proliferation of HeLa cells but not HMC cells, measured by MTT assay. 1 × 10^5^ of HeLa cells or 1 × 10^5^ of HMC cells was cultured either in media, or plus different concentrations (25, 20, 15, 10, and 5 μg/ml) of caerin 1.1, caerin 1.9, the mixture of 1.1 and 1.9 (mass ratio 1 : 1) or P3 for 24 hours before MTT assay was performed. (A) Caerin 1.1 treated HeLa cells, (B) caerin 1.9 treated HeLa cells, (C) the mixture treated HeLa cells, (D) P3 treated HeLa cells, (E) caerin 1.1 treated HMC cells, (F) caerin 1.9 treated HMC, (G) the mixture treated HMC cells, and (H) P3 treated HMC cells. Each bar represents the statistical mean from three biological replicates (performed in triplicate) and the error bars represent the standard deviation. Figure S2: probabilistic PCA is used to calculate principal components. x and y-axes show principal component 1 and principal component 2 that explain 68.8% and 19.5% of the total variance, respectively. N = 9 data points. Figure S3: heatmap of differentially expressed proteins in TC-1 cells identified from iTRAQ analysis of the first biological replicate treated with caerin 1.9 and the mixture (caerin 1.9 plus caerin 1.1 at a mass ratio of 1 : 1) at 24 h and untreated cells as control. The figure was generated using PEAKS studio. The decreased and increased proteins are indicated by range of green and red intensities, respectively. Figure S4: heatmap of differentially expressed proteins in TC-1 cells identified from iTRAQ analysis of the second biological replicate treated with caerin 1.9 and the mixture (caerin 1.9 plus caerin 1.1 at a mass ratio of 1 : 1) at 24 h and untreated cells as control. The figure was generated using PEAKS studio. The decreased and increased proteins are indicated by range of green and red intensities, respectively. Figure S5: heatmap of differentially expressed proteins in TC-1 cells identified from iTRAQ analysis of the third biological replicat [file 7382351.f1.zip › S1 File.pdf]
