# Supplementary figures and images for: Comparative Proteomic Study of the Antiproliferative Activity of Frog Host-Defence Peptide Caerin 1.9 and Its Additive Effect with Caerin 1.1 on TC-1 Cells Transformed with HPV16 E6 and E7
Source: Biomed Res Int. 2018 May 13;2018:7382351. doi: 10.1155/2018/7382351 (PMC5971270; doi:10.1155/2018/7382351)

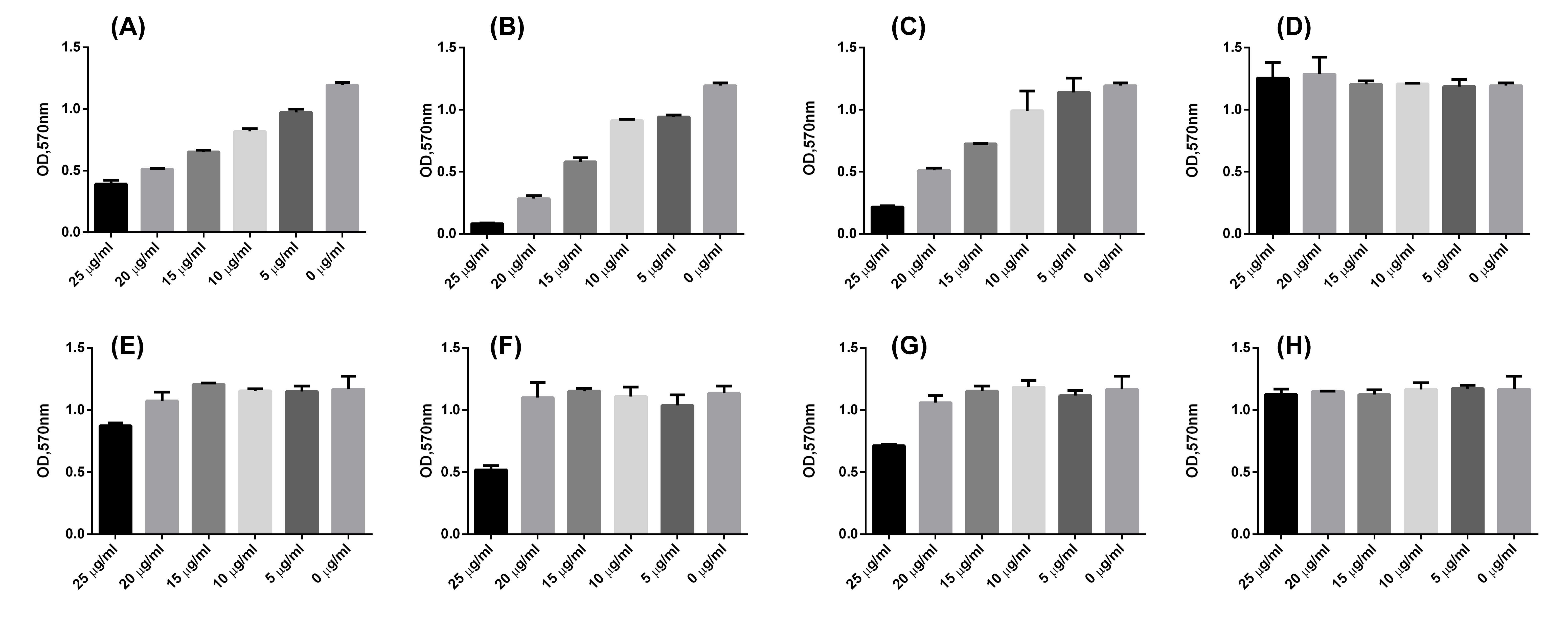

Supplement: Supplementary Materials — Figure S1: caerin 1.1 and caerin 1.9 are able to inhibit the proliferation of HeLa cells but not HMC cells, measured by MTT assay. 1 × 10^5^ of HeLa cells or 1 × 10^5^ of HMC cells was cultured either in media, or plus different concentrations (25, 20, 15, 10, and 5 μg/ml) of caerin 1.1, caerin 1.9, the mixture of 1.1 and 1.9 (mass ratio 1 : 1) or P3 for 24 hours before MTT assay was performed. (A) Caerin 1.1 treated HeLa cells, (B) caerin 1.9 treated HeLa cells, (C) the mixture treated HeLa cells, (D) P3 treated HeLa cells, (E) caerin 1.1 treated HMC cells, (F) caerin 1.9 treated HMC, (G) the mixture treated HMC cells, and (H) P3 treated HMC cells. Each bar represents the statistical mean from three biological replicates (performed in triplicate) and the error bars represent the standard deviation. Figure S2: probabilistic PCA is used to calculate principal components. x and y-axes show principal component 1 and principal component 2 that explain 68.8% and 19.5% of the total variance, respectively. N = 9 data points. Figure S3: heatmap of differentially expressed proteins in TC-1 cells identified from iTRAQ analysis of the first biological replicate treated with caerin 1.9 and the mixture (caerin 1.9 plus caerin 1.1 at a mass ratio of 1 : 1) at 24 h and untreated cells as control. The figure was generated using PEAKS studio. The decreased and increased proteins are indicated by range of green and red intensities, respectively. Figure S4: heatmap of differentially expressed proteins in TC-1 cells identified from iTRAQ analysis of the second biological replicate treated with caerin 1.9 and the mixture (caerin 1.9 plus caerin 1.1 at a mass ratio of 1 : 1) at 24 h and untreated cells as control. The figure was generated using PEAKS studio. The decreased and increased proteins are indicated by range of green and red intensities, respectively. Figure S5: heatmap of differentially expressed proteins in TC-1 cells identified from iTRAQ analysis of the third biological replicat [file 7382351.f1.zip › S1 Fig.png]

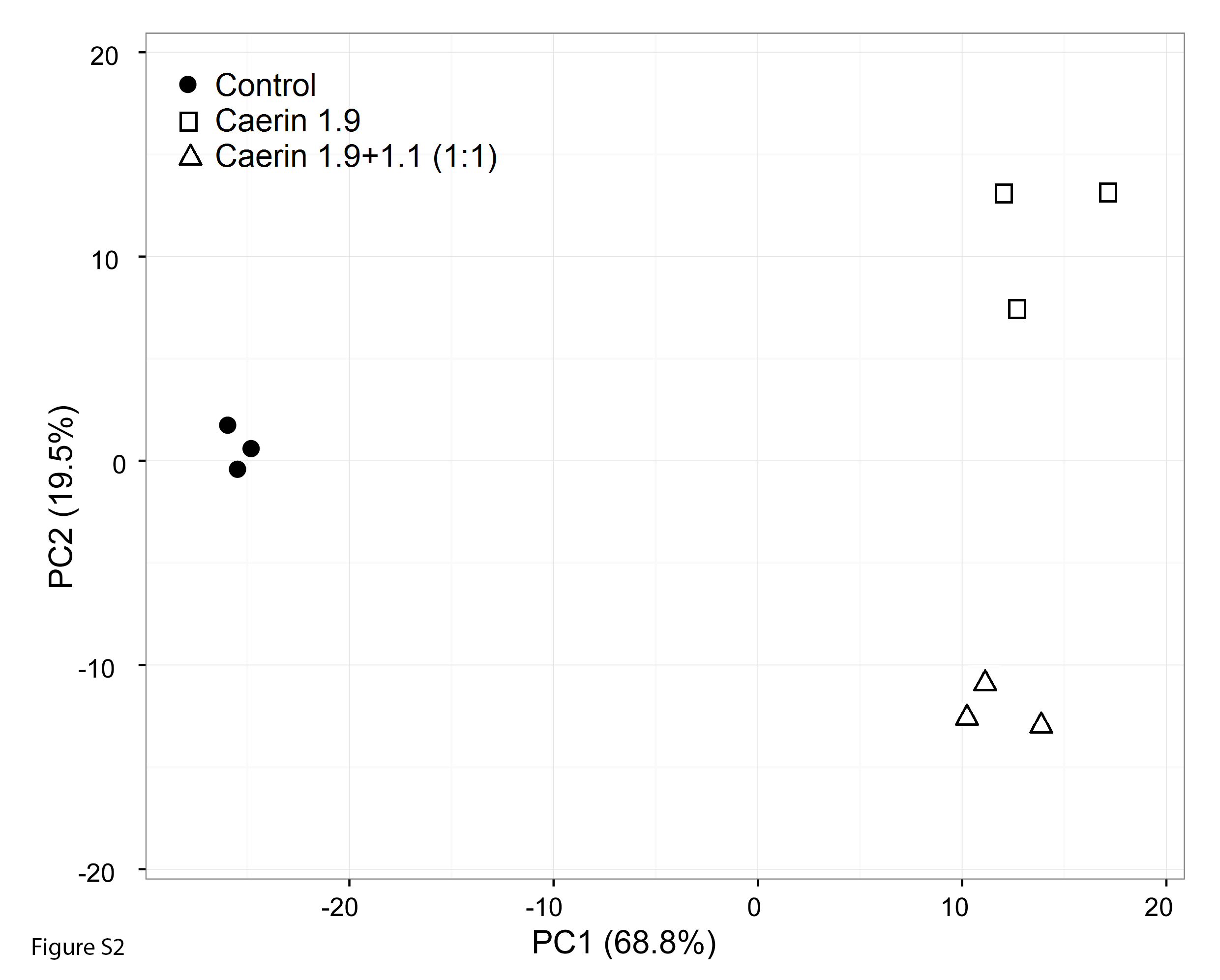

Supplement: Supplementary Materials — Figure S1: caerin 1.1 and caerin 1.9 are able to inhibit the proliferation of HeLa cells but not HMC cells, measured by MTT assay. 1 × 10^5^ of HeLa cells or 1 × 10^5^ of HMC cells was cultured either in media, or plus different concentrations (25, 20, 15, 10, and 5 μg/ml) of caerin 1.1, caerin 1.9, the mixture of 1.1 and 1.9 (mass ratio 1 : 1) or P3 for 24 hours before MTT assay was performed. (A) Caerin 1.1 treated HeLa cells, (B) caerin 1.9 treated HeLa cells, (C) the mixture treated HeLa cells, (D) P3 treated HeLa cells, (E) caerin 1.1 treated HMC cells, (F) caerin 1.9 treated HMC, (G) the mixture treated HMC cells, and (H) P3 treated HMC cells. Each bar represents the statistical mean from three biological replicates (performed in triplicate) and the error bars represent the standard deviation. Figure S2: probabilistic PCA is used to calculate principal components. x and y-axes show principal component 1 and principal component 2 that explain 68.8% and 19.5% of the total variance, respectively. N = 9 data points. Figure S3: heatmap of differentially expressed proteins in TC-1 cells identified from iTRAQ analysis of the first biological replicate treated with caerin 1.9 and the mixture (caerin 1.9 plus caerin 1.1 at a mass ratio of 1 : 1) at 24 h and untreated cells as control. The figure was generated using PEAKS studio. The decreased and increased proteins are indicated by range of green and red intensities, respectively. Figure S4: heatmap of differentially expressed proteins in TC-1 cells identified from iTRAQ analysis of the second biological replicate treated with caerin 1.9 and the mixture (caerin 1.9 plus caerin 1.1 at a mass ratio of 1 : 1) at 24 h and untreated cells as control. The figure was generated using PEAKS studio. The decreased and increased proteins are indicated by range of green and red intensities, respectively. Figure S5: heatmap of differentially expressed proteins in TC-1 cells identified from iTRAQ analysis of the third biological replicat [file 7382351.f1.zip › S2 Fig.png]

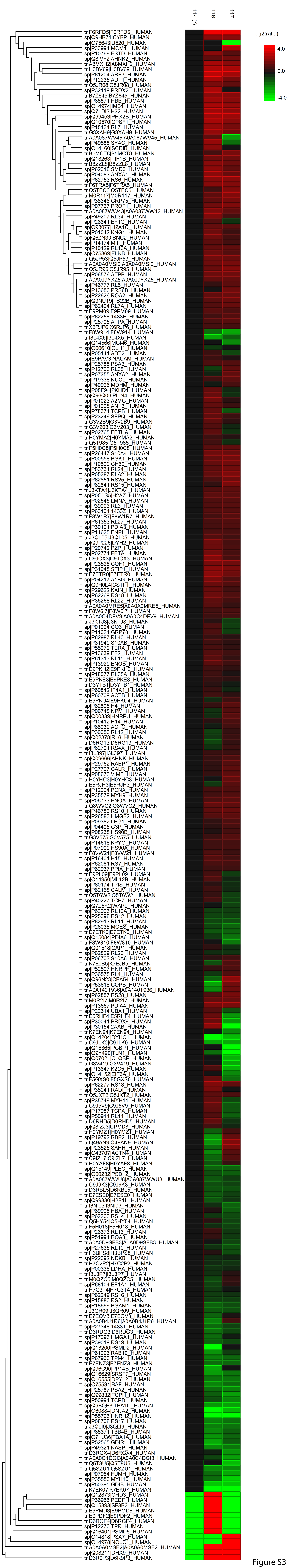

Supplement: Supplementary Materials — Figure S1: caerin 1.1 and caerin 1.9 are able to inhibit the proliferation of HeLa cells but not HMC cells, measured by MTT assay. 1 × 10^5^ of HeLa cells or 1 × 10^5^ of HMC cells was cultured either in media, or plus different concentrations (25, 20, 15, 10, and 5 μg/ml) of caerin 1.1, caerin 1.9, the mixture of 1.1 and 1.9 (mass ratio 1 : 1) or P3 for 24 hours before MTT assay was performed. (A) Caerin 1.1 treated HeLa cells, (B) caerin 1.9 treated HeLa cells, (C) the mixture treated HeLa cells, (D) P3 treated HeLa cells, (E) caerin 1.1 treated HMC cells, (F) caerin 1.9 treated HMC, (G) the mixture treated HMC cells, and (H) P3 treated HMC cells. Each bar represents the statistical mean from three biological replicates (performed in triplicate) and the error bars represent the standard deviation. Figure S2: probabilistic PCA is used to calculate principal components. x and y-axes show principal component 1 and principal component 2 that explain 68.8% and 19.5% of the total variance, respectively. N = 9 data points. Figure S3: heatmap of differentially expressed proteins in TC-1 cells identified from iTRAQ analysis of the first biological replicate treated with caerin 1.9 and the mixture (caerin 1.9 plus caerin 1.1 at a mass ratio of 1 : 1) at 24 h and untreated cells as control. The figure was generated using PEAKS studio. The decreased and increased proteins are indicated by range of green and red intensities, respectively. Figure S4: heatmap of differentially expressed proteins in TC-1 cells identified from iTRAQ analysis of the second biological replicate treated with caerin 1.9 and the mixture (caerin 1.9 plus caerin 1.1 at a mass ratio of 1 : 1) at 24 h and untreated cells as control. The figure was generated using PEAKS studio. The decreased and increased proteins are indicated by range of green and red intensities, respectively. Figure S5: heatmap of differentially expressed proteins in TC-1 cells identified from iTRAQ analysis of the third biological replicat [file 7382351.f1.zip › S3 Fig.png]

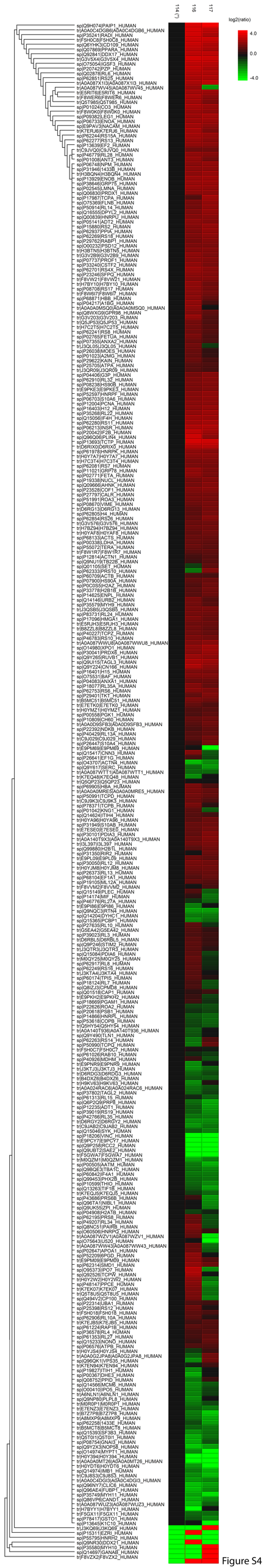

Supplement: Supplementary Materials — Figure S1: caerin 1.1 and caerin 1.9 are able to inhibit the proliferation of HeLa cells but not HMC cells, measured by MTT assay. 1 × 10^5^ of HeLa cells or 1 × 10^5^ of HMC cells was cultured either in media, or plus different concentrations (25, 20, 15, 10, and 5 μg/ml) of caerin 1.1, caerin 1.9, the mixture of 1.1 and 1.9 (mass ratio 1 : 1) or P3 for 24 hours before MTT assay was performed. (A) Caerin 1.1 treated HeLa cells, (B) caerin 1.9 treated HeLa cells, (C) the mixture treated HeLa cells, (D) P3 treated HeLa cells, (E) caerin 1.1 treated HMC cells, (F) caerin 1.9 treated HMC, (G) the mixture treated HMC cells, and (H) P3 treated HMC cells. Each bar represents the statistical mean from three biological replicates (performed in triplicate) and the error bars represent the standard deviation. Figure S2: probabilistic PCA is used to calculate principal components. x and y-axes show principal component 1 and principal component 2 that explain 68.8% and 19.5% of the total variance, respectively. N = 9 data points. Figure S3: heatmap of differentially expressed proteins in TC-1 cells identified from iTRAQ analysis of the first biological replicate treated with caerin 1.9 and the mixture (caerin 1.9 plus caerin 1.1 at a mass ratio of 1 : 1) at 24 h and untreated cells as control. The figure was generated using PEAKS studio. The decreased and increased proteins are indicated by range of green and red intensities, respectively. Figure S4: heatmap of differentially expressed proteins in TC-1 cells identified from iTRAQ analysis of the second biological replicate treated with caerin 1.9 and the mixture (caerin 1.9 plus caerin 1.1 at a mass ratio of 1 : 1) at 24 h and untreated cells as control. The figure was generated using PEAKS studio. The decreased and increased proteins are indicated by range of green and red intensities, respectively. Figure S5: heatmap of differentially expressed proteins in TC-1 cells identified from iTRAQ analysis of the third biological replicat [file 7382351.f1.zip › S4 Fig.png]

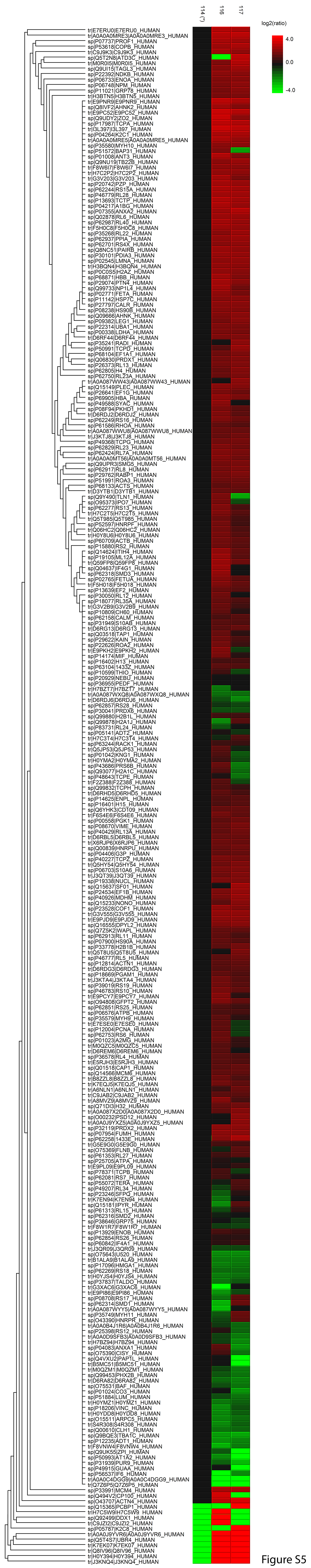

Supplement: Supplementary Materials — Figure S1: caerin 1.1 and caerin 1.9 are able to inhibit the proliferation of HeLa cells but not HMC cells, measured by MTT assay. 1 × 10^5^ of HeLa cells or 1 × 10^5^ of HMC cells was cultured either in media, or plus different concentrations (25, 20, 15, 10, and 5 μg/ml) of caerin 1.1, caerin 1.9, the mixture of 1.1 and 1.9 (mass ratio 1 : 1) or P3 for 24 hours before MTT assay was performed. (A) Caerin 1.1 treated HeLa cells, (B) caerin 1.9 treated HeLa cells, (C) the mixture treated HeLa cells, (D) P3 treated HeLa cells, (E) caerin 1.1 treated HMC cells, (F) caerin 1.9 treated HMC, (G) the mixture treated HMC cells, and (H) P3 treated HMC cells. Each bar represents the statistical mean from three biological replicates (performed in triplicate) and the error bars represent the standard deviation. Figure S2: probabilistic PCA is used to calculate principal components. x and y-axes show principal component 1 and principal component 2 that explain 68.8% and 19.5% of the total variance, respectively. N = 9 data points. Figure S3: heatmap of differentially expressed proteins in TC-1 cells identified from iTRAQ analysis of the first biological replicate treated with caerin 1.9 and the mixture (caerin 1.9 plus caerin 1.1 at a mass ratio of 1 : 1) at 24 h and untreated cells as control. The figure was generated using PEAKS studio. The decreased and increased proteins are indicated by range of green and red intensities, respectively. Figure S4: heatmap of differentially expressed proteins in TC-1 cells identified from iTRAQ analysis of the second biological replicate treated with caerin 1.9 and the mixture (caerin 1.9 plus caerin 1.1 at a mass ratio of 1 : 1) at 24 h and untreated cells as control. The figure was generated using PEAKS studio. The decreased and increased proteins are indicated by range of green and red intensities, respectively. Figure S5: heatmap of differentially expressed proteins in TC-1 cells identified from iTRAQ analysis of the third biological replicat [file 7382351.f1.zip › S5 Fig.png]

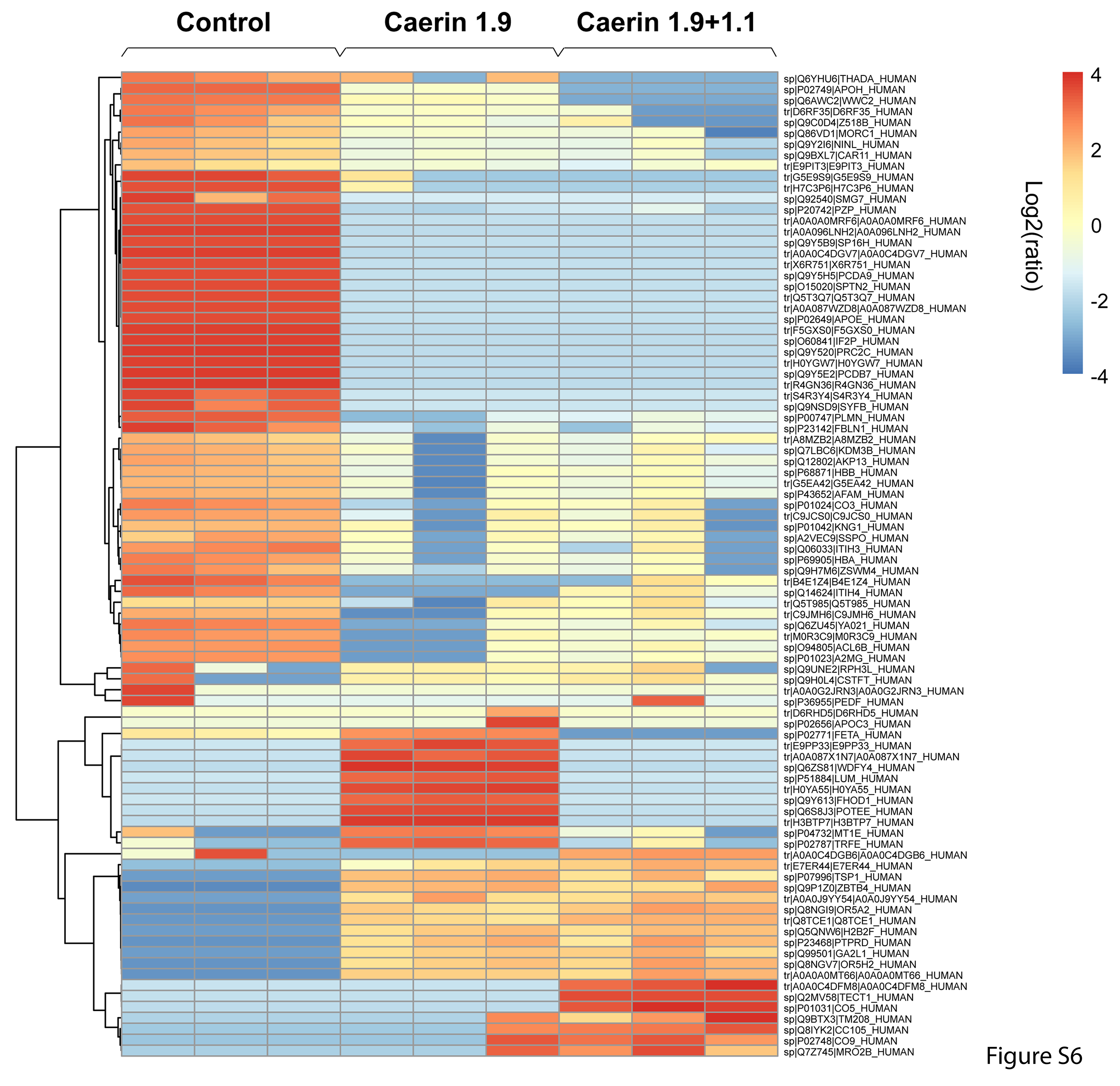

Supplement: Supplementary Materials — Figure S1: caerin 1.1 and caerin 1.9 are able to inhibit the proliferation of HeLa cells but not HMC cells, measured by MTT assay. 1 × 10^5^ of HeLa cells or 1 × 10^5^ of HMC cells was cultured either in media, or plus different concentrations (25, 20, 15, 10, and 5 μg/ml) of caerin 1.1, caerin 1.9, the mixture of 1.1 and 1.9 (mass ratio 1 : 1) or P3 for 24 hours before MTT assay was performed. (A) Caerin 1.1 treated HeLa cells, (B) caerin 1.9 treated HeLa cells, (C) the mixture treated HeLa cells, (D) P3 treated HeLa cells, (E) caerin 1.1 treated HMC cells, (F) caerin 1.9 treated HMC, (G) the mixture treated HMC cells, and (H) P3 treated HMC cells. Each bar represents the statistical mean from three biological replicates (performed in triplicate) and the error bars represent the standard deviation. Figure S2: probabilistic PCA is used to calculate principal components. x and y-axes show principal component 1 and principal component 2 that explain 68.8% and 19.5% of the total variance, respectively. N = 9 data points. Figure S3: heatmap of differentially expressed proteins in TC-1 cells identified from iTRAQ analysis of the first biological replicate treated with caerin 1.9 and the mixture (caerin 1.9 plus caerin 1.1 at a mass ratio of 1 : 1) at 24 h and untreated cells as control. The figure was generated using PEAKS studio. The decreased and increased proteins are indicated by range of green and red intensities, respectively. Figure S4: heatmap of differentially expressed proteins in TC-1 cells identified from iTRAQ analysis of the second biological replicate treated with caerin 1.9 and the mixture (caerin 1.9 plus caerin 1.1 at a mass ratio of 1 : 1) at 24 h and untreated cells as control. The figure was generated using PEAKS studio. The decreased and increased proteins are indicated by range of green and red intensities, respectively. Figure S5: heatmap of differentially expressed proteins in TC-1 cells identified from iTRAQ analysis of the third biological replicat [file 7382351.f1.zip › S6 Fig.png]
